# Supplementary material for: Strong anion exchange‐mediated phosphoproteomics reveals extensive human non‐canonical phosphorylation
Source: EMBO J. 2019 Aug 21;38(21):e100847. doi: 10.15252/embj.2018100847 (PMC6826212; doi:10.15252/embj.2018100847)
Supplement: Supplementary file 1 — Appendix [file EMBJ-38-e100847-s001.pdf]

Appendix: Strong anion exchange-mediated phosphoproteomics reveals extensive human non-canonical phosphorylation

Gemma Hardman[1§], Simon Perkins[2§], Philip Brownridge[1], Christopher J. Clarke[1], Dominic P. Byrne[3], Amy E. Campbell[1], Anton Kalyuzhnyy[2], Ashleigh Myall[2], Patrick A. Eyers[3], Andrew R. Jones[2] and Claire E. Eyers[1]*

[1] Centre for Proteome Research, Department of Biochemistry, Institute of Integrative Biology, University of Liverpool, Crown Street, Liverpool, L69 7ZB, UK
[2] Department of Comparative and Functional Genomics, Institute of Integrative Biology, University of Liverpool, Crown Street, Liverpool, L69 7ZB, UK
[3] Department of Biochemistry, Institute of Integrative Biology, University of Liverpool, Crown Street, Liverpool, L69 7ZB, UK

[§] Both authors contributed equally to this work

Table of Contents

Supplementary Methods ***3***

Phosphohistidine dot blot *3*

Phosphohistidine peptide stability test *3*

Calcium phosphate precipitation *3*

Hydroxyapatite enrichment *3*

Intact protein MS analysis *4*

Estimation of false localisation rate (FLR) *4*

Appendix Figures ***6***

Appendix Figure S1. Stability profiles of pHis-containing tryptic peptides. *6*

Appendix Figure S2. pHis-containing peptides undergo hydrolysis during TiO2 enrichment. *7*

Appendix Figure S3. Numbers of non-phosphorylated and phosphorylated peptides identified per pooled SAX fraction (5% FDR), according to phosphorylated amino acid (ptmRS ≥0.90). *8*

Appendix Figure S4. ptmRS distribution (probability) of peptide spectrum matches (PSMs) according to phosphorylated residue. *9*

Appendix Figure S5. pAla computed FLR estimate for each type of canonical and non-canonical phosphorylated residue *10*

Appendix Figure S6. Example tandem mass spectra for pHis-containing peptides *11*

Appendix Figure S7. Spectral comparison of HCD tandem mass spectra of synthetic chemically phosphorylated pHis peptides with the analogous pHis-containing spectra from the high-throughput UPAX data. *12*

Appendix Figure S8. Motif analysis for pHis-containing peptides. *14*

Appendix Figure S9. EThcD product ion mass spectrum of singly phosphorylated DHSPTPSVFNSDEER from FIP1L1. *15*

Appendix Figure S10. Example tandem mass spectra for pAsp-containing peptides. *16*

Appendix Figure S11. Motif analysis for pAsp-containing peptides. *17*

Appendix Figure S12. Example tandem mass spectra for pGlu-containing peptides. *18*

Appendix Figure S13. Motif analysis for pGlu-containing peptides. *19*

Appendix Figure S14. Example tandem mass spectra for pLys-containing peptides. *20*

Appendix Figure S15. Motif analysis for pLys-containing peptides. *21*

Appendix Figure S16. Example tandem mass spectra for pArg-containing peptides. *22*

Appendix Figure S17. Motif analysis for pArg-containing peptides. *23*

Appendix Figure S18. Motif analysis for pCys-containing peptides. *24*

Appendix Tables ***25***

Appendix Table S1. Phosphohistidine-containing tryptic peptides from myoglobin. *25*

Appendix Table S2. Conditions evaluated for TiO_2 enrichment of pHis (and other) phosphopeptides. *25*

Appendix Table S3. Enrichment of pHis peptides by TiO_2 fails due to acid-induced hydrolysis of pHis. *26*

Appendix Table S4. Recovery of pHis peptides following hydroxyapatite chromatography. *26*

Appendix Table S5. Total number of phosphopeptides and unique sites identified (5% PSM FDR) *27*

Appendix Table S6. Proportion of phosphorylated residues identified using either UPAX or with a standard TiO_2-based phosphopeptide enrichment workflow. *28*

Appendix Table S7. Total number of phosphopeptides and unique sites identified (1% PSM FDR) *29*

Appendix Table S8. Worked example demonstrating how False Localisation Rate (FLR) is estimated at ptmRS ≥ 0.90 based on the number of pAla 'identifications'. *30*

Appendix Table S9. Phosphoimmonium ions are not generally indicative of the phosphorylated residue. *31*

References ***32***

Supplementary Methods

Phosphohistidine dot blot

Recombinant PGAM and NME1 proteins (Clubbs-Couldron *et al., in press*) were histidine-phosphorylated *in vitro* by incubation with 1 mM 2,3-diphosphoglycerate (DPG, Sigma) or 1 mM adenosine triphosphate (ATP, Sigma), respectively, for 5 min at room temperature. Myoglobin was phosphorylated with potassium phosphoramidate as described in Methods. All proteins (phosphorylated and unphosphorylated) were then dotted onto nitrocellulose membrane (GE Healthcare) and allowed to dry completely. Membranes were blocked with 5% (w/v) non-fat dry milk (Marvel) in TBS with 0.1% (v/v) Tween20, and incubated overnight at 4°C with either N1-phosphohistidine antibody (Merck MABS1341, clone SC50-3), N3-phosphohistidine antibody (Merck MABS1351, clone SC39-6) or no antibody (Fuhs *et al.* 2015). The next day, all membranes were incubated with an HRP-conjugated anti-mouse secondary antibody (Cell Signaling Technology, #7076) and imaged on a ChemiDoc MP Imaging System (BioRad).

Phosphohistidine peptide stability test

Myoglobin peptides (1 nmol) were diluted to 100 µL in either 0.5% TFA (pH 1) or 20 mM ammonium acetate (pH 4, pH 6 or pH 9). Samples were incubated at 25 °C with shaking at 600 rpm. At timed intervals (15 minutes, 30 minutes, 1 hour and 2 hours) 5 µL of sample was removed, neutralised (e.g. by addition of 5 µL ammonium hydroxide to samples at pH 1) and diluted to 500 fmol/µL with H_2O:ACN (97:3) for LC-MS/MS analysis with the Bruker AmaZon instrument. This experiment was performed in triplicate for each pH.

Calcium phosphate precipitation

Myoglobin and α/β-casein peptides (50 pmol) were diluted to 50 µL in H_2O. Sodium phosphate (2 µL of 0.5 M) and ammonia water (2 µL of 2 M) were added, and the pH of the resulting solution determined to be approximately pH 10 using universal indicator paper. Phosphopeptides were enriched using calcium phosphoate precipitation according to the method described in (Zhang *et al* 2007). $CaCl_2$ (2 µL of 2 M) was added and the sample mixed using a vortex mixer for 5 minutes, followed by centrifugation at 13,000 rpm for 15 minutes. The resulting supernatant was transferred to a clean low bind sample tube. $CaCl_2$ (100 µL of 80 mM) was added to the precipitate which was briefly mixed and then centrifuged as before. The resulting supernatant was combined with that of the previous step, and the wash step repeated with a further 100 µL of 80 mM $CaCl_2$, again combining the supernatant fractions. The precipitate was resolubilised in 10 µL of either 5% (v/v) or 0.1% (v/v) TFA and immediately transferred to a C18 StageTip for sample desalting.

Hydroxyapatite enrichment

Phosphopeptides were enriched according using a method adapted from (Mamone *et* al 2010). Hydroxyapatite (HAP) resin (5 mg; Bio-Gel HTP, Bio-Rad) was suspended in 200 µL loading buffer (20 mM Tris-HCl (pH 7.2)) and added to a Pierce spin column (Thermo Scientific). This was centrifuged at 300 *g* for 1 minute to remove the buffer and washed with a further 50 µL loading buffer, and again centrifuged. Centrifugation of the spin column at 3000 *g* for 1 minute was used to collect all subsequent fractions. Tryptic peptides of α- and β-casein and histidine-phosphorylated myoglobin (40 pmol) in 50 µL of loading buffer was added to the resin in the spin column and incubated with gentle rotation at room temperature for 30-45 minutes. The flow-through was collected and the resin washed twice with 200 µL of loading buffer, which was also collected. The resin was then washed twice with 200 µL of wash buffer (20 mM Tris-HCl (pH 7.2), 20% (v/v) ACN) which was again collected. To recover the phosphopeptides, the resin was incubated twice with 100 µL elution buffer (1.0 M K_2HPO_4 at pH 7.8, pH 7.0 or pH 6.8), rotating for 15

minutes at room temperature each time, before collecting and combining the eluent fractions. All fractions were dried by vacuum centrifugation and reconstituted in H_2O:ACN (97:3) for LC-MS/MS analysis with the Bruker AmaZon instrument. As an alternative approach tryptic peptides of α- and β-casein and histidine-phosphorylated myoglobin (20 pmol) in 50 µL of loading buffer was added to HAP resin and washed as described above. The resin was then washed with H_2O (100 µL) and incubated with 500 mM hydroxylamine (50 µL) for 1 hour at 37 °C with shaking. This was collected and all eluates were dried and analysed as previously described.

Intact protein MS analysis

Analysis of intact phosphorylated myoglobin was conducted using a Waters Synapt G2-S*i* instrument. Phosphorylated myoglobin (5 µM in 20 mM ammonium acetate:ACN 50:50) was analysed using borosilicate emitters (Thermo ES 387). Spraying voltage was adjusted to 1.5 kV, sampling cone was 50 V. The time-of-flight mass analyser was set to Resolution Mode. Data was processed using Mass Lynx V4.1, with deconvolution performed using MaxEnt 1.

Estimation of false localisation rate (FLR)

To address a potential concern that consideration of multiple phosphorylated residues was compromising statistical confidence in phosphosite identification, we used the same search parameters to evaluate the prevalence of a theoretical pAla residue, by substituting variable modification of pCys for pAla. It was first verified that the "pAla" search results produced almost identical counts of PSMs at 5% FDR. We analysed the results from the ptmRS scoring of pAla at different thresholds to estimate the FLR amongst the different residues in the main search. Identifications of pAla are known to be chemically impossible, so we use apparent pAla counts (at different ptmRS thresholds) to estimate the rate at which the workflow *randomly* assigns a phosphosite to a given amino acid. The pAla counts thus need to be normalised per amino acid considered, taking into account the relative frequencies of each amino acid i.e. a rare amino acid would have fewer chances of being randomly (and wrongly) assigned a phosphosite than a common amino acid.

To normalise pAla counts at a given threshold, we first calculated the count of each amino acid within the set of redundant PSMs supporting phosphopeptides only. The rationale for normalising first by amino acid counts within the redundant phosphopeptide set considers two factors. First, only identified phosphopeptides progress to ptmRS scoring, so it is less relevant what amino acid counts are present in the search database or in peptides without phosphosites. Particular amino acids are seen more or less than would be expected by chance in the set of phosphopeptides e.g. Glu residues appear only 10% more than Ala in the search database, but more than 2X higher in the PSMs supporting phosphopeptides, and thus FLR estimates need to reflect this fact. Second, normalisation first in the redundant (non-unique) set is likely to be more conservative, if there are particular amino acids enriched on (abundant) peptides supported by many PSMs, would give more opportunities for ptmRS to assign false localisations to these amino acids.

We calculated the count of non-unique pAla sites at each given ptmRS threshold NU_S_{pAla}, and the count of all Ala residues NU_PSMs_{Ala} in the non-unique PSM set. We then calculated the frequency of any amino acid being randomly assigned a phosphorylation site: $AA\_RF = NU\_S_{pAla} / NU\_PSMs_{Ala}$. To estimate the non-unique false localisation site count per amino acid, we used the following formula: $NU\_FLS_x = AA\_RF * NU\_PSMs_x$ (where x = C, D, E, H, K non C-terminal, R non C-terminal, S, T or Y). As noted in the text, we observe unexpectedly high counts of C-terminal sites on Lys and Arg, so for FLR calculation, only non C-terminal sites are considered.

We next calculated the FLR within the unique set of sites by estimating a single *collapse factor* (*CF)* to model the rate at which random false positive sites collapse from the non-unique counts to unique site counts, as follows $CF = U\_S_{pAla} / NU\_S_{pAla}$. For each amino acid, the final estimate of the unique false localisation site Count $U\_FLS_x = NU\_FLS_x * CF$ and the final unique false localisation rate $FLR_x = U\_FLS_x / U\_S_x$ *(where* U_S_x *is the count of unique sites per amino acid).* The rationale for using a single collapse factor for all residues is that it would be expected that false localisations would collapse from the non-unique to the unique-level at a different rate i.e. true positive sites would be predicted to be, on average, supported by more PSMs than random, incorrect sites. Thus we use the CF estimate from pAla only to estimate the conversion from non-unique to unique false positive sites. For each amino acid, the estimate of true positive sites is $TPS_x = U\_S_x – U\_FLS_x$. See Appendix Table S5 for a worked example at *ptm*RS > 0.90, where NU_PSMs_{Ala} = ***51205***; NU_S_{pAla} = ***263****, therefore AA_RF* = 0.005136.

Appendix Figures

A

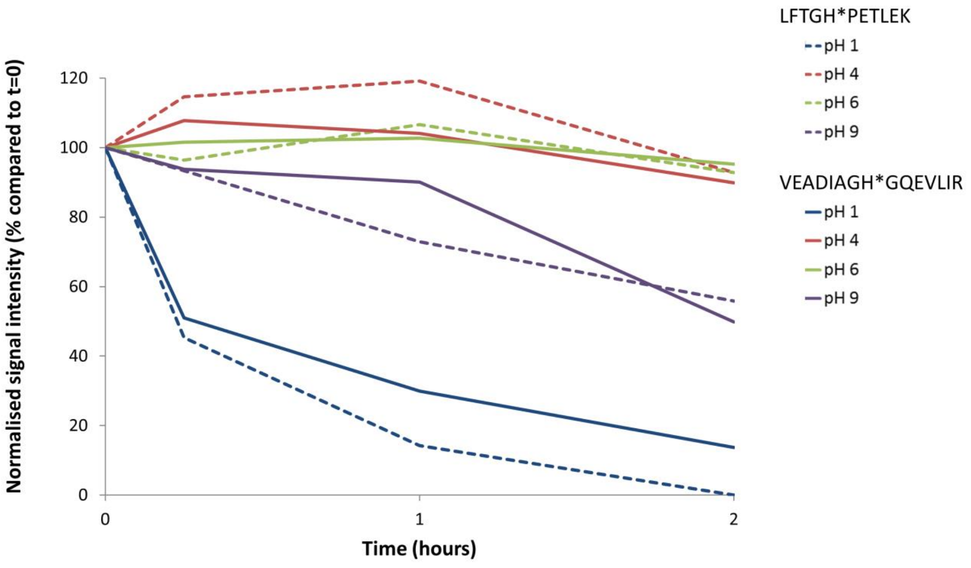

B

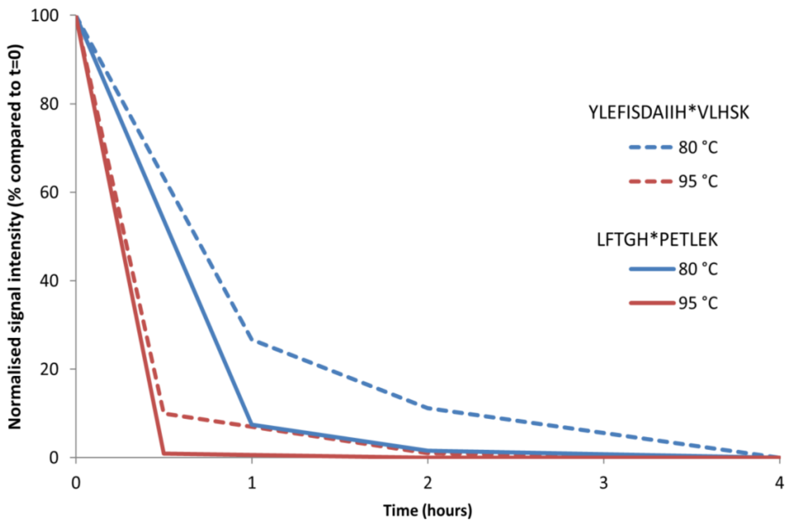

Appendix Figure S1. Stability profiles of pHis-containing tryptic peptides.

Extracted ion chromatograms were used to determine the normalised relative signal intensity of the pHis-containing tryptic myoglobin peptides LFTGH*PETLEK at *m/z* 676.3 and VEADIAGH*GQEVLIR at *m/z* 843.9 as a function of (**A**) pH at pH 9, pH 6, pH 4 and pH 1, or (B) temperature at either 80 °C or 95 °C at pH 7.2. Signal intensity was normalised against a non-histidine-containing peptide and presented as a percentage compared to the amount at time t=0. Phosphohistidine-containing peptides exhibit a much reduced stability at pH 1 compared to pH 4, pH 6 and pH 9. Data is representative of all myoglobin pHis-containing peptides.

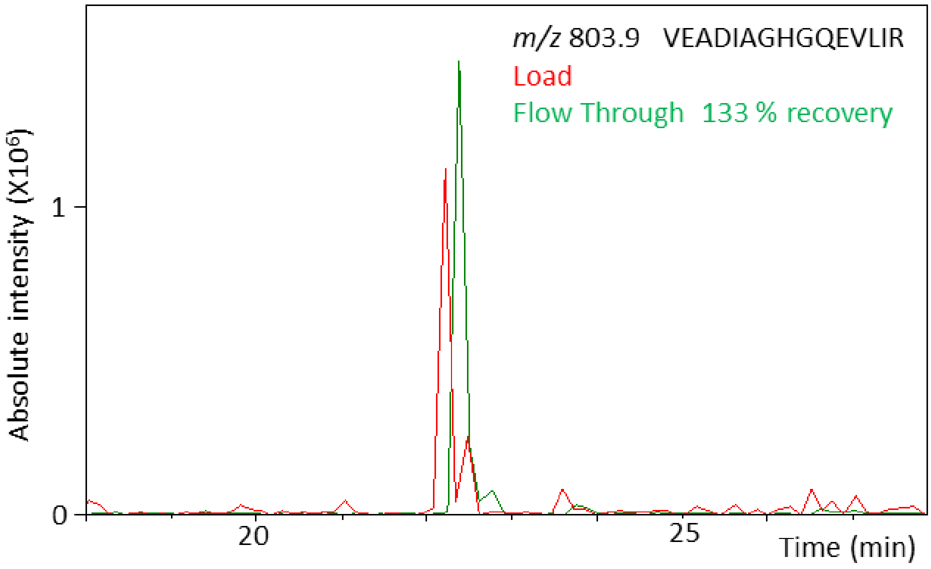

Appendix Figure S2. pHis-containing peptides undergo hydrolysis during TiO_2 enrichment.
Overlay of extracted ion chromatograms at *m/z* 803.9 for the non-phosphorylated counterpart of the pHis-containing myoglobin peptide VEADIAGHGQEVLIR before (load; red) and after (recovery; green) TiO_2 phosphopeptide enrichment under standard acidic conditions. Data is representative of multiple pHis peptides and multiple repeats. >100% recovery of the non-phosphorylated peptide indicates neutral loss of phosphate from the corresponding pHis-containing peptide.

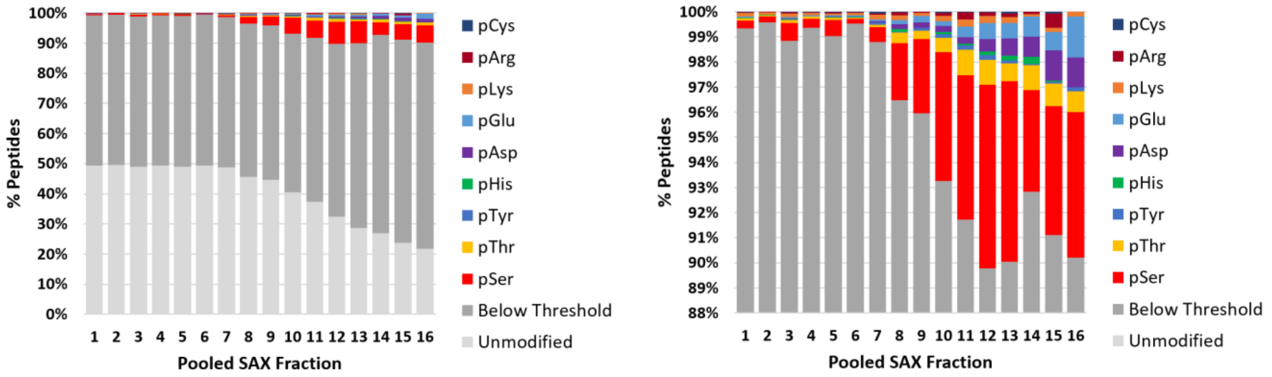

Appendix Figure S3. Numbers of non-phosphorylated and phosphorylated peptides identified per pooled SAX fraction (5% FDR), according to phosphorylated amino acid (ptmRS ≥0.90).

Below threshold refers to phosphopeptides with ptmRS < 0.90. Phosphopeptide diversity is highlighted in the zoomed region in the right panel.

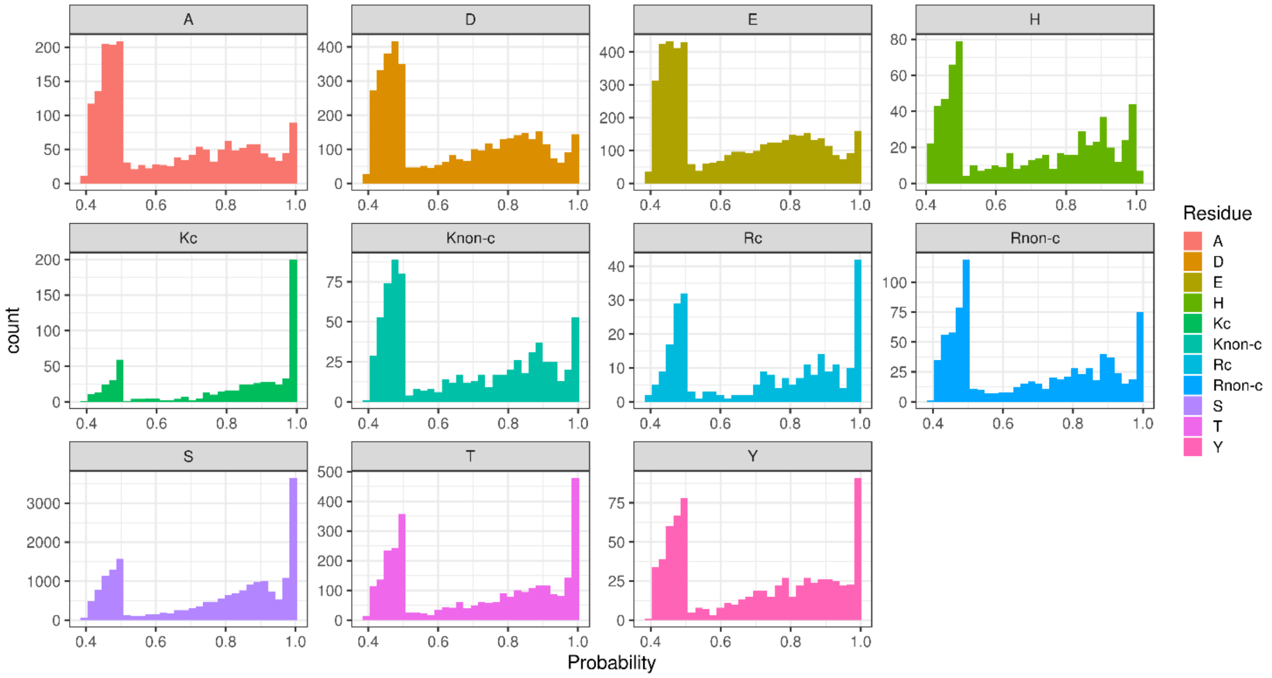

Appendix Figure S4. *ptm*RS distribution (probability) of peptide spectrum matches (PSMs) according to phosphorylated residue.

The number of PSMs assigned as a being phosphorylated on Ala, Asp, Glu, His, Lys (localised either to the peptide C-terminus (Kc), or internally (Knon-c)), Arg (Rc or Rnon-C), Ser, Thr or Tyr are binned according to probability of site localisation (*ptm*RS) score (0.4 to 1.0). Score distribution for pAla is broadly similar to that observed for other (non-C-terminally assigned) phosphorylated residues.

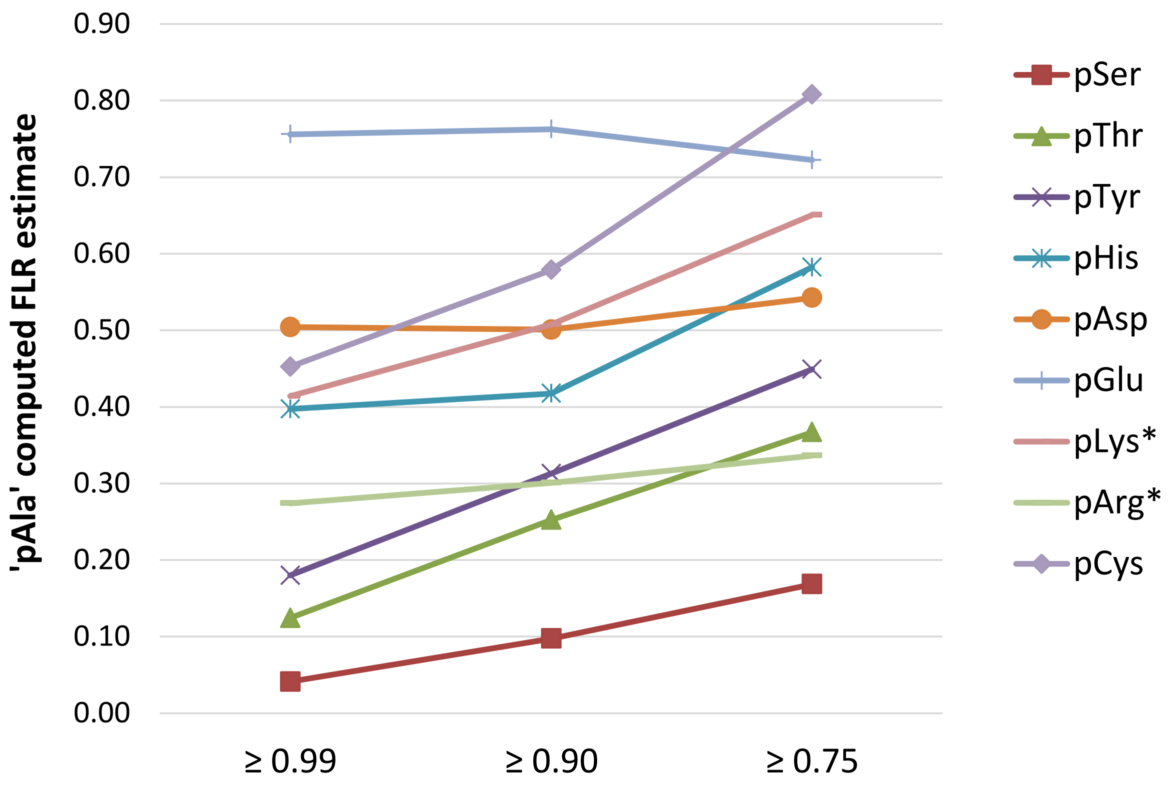

Appendix Figure S5. pAla computed FLR estimate for each type of canonical and non-canonical phosphorylated residue at ptmRS values ≥0.99, ≥0.90 or ≥0.75 at a 1% PSM FDR. pLys* and pArg* represent those phosphorylation sites on Lys or Arg (respectively) that are not localised to the peptide C-terminus.

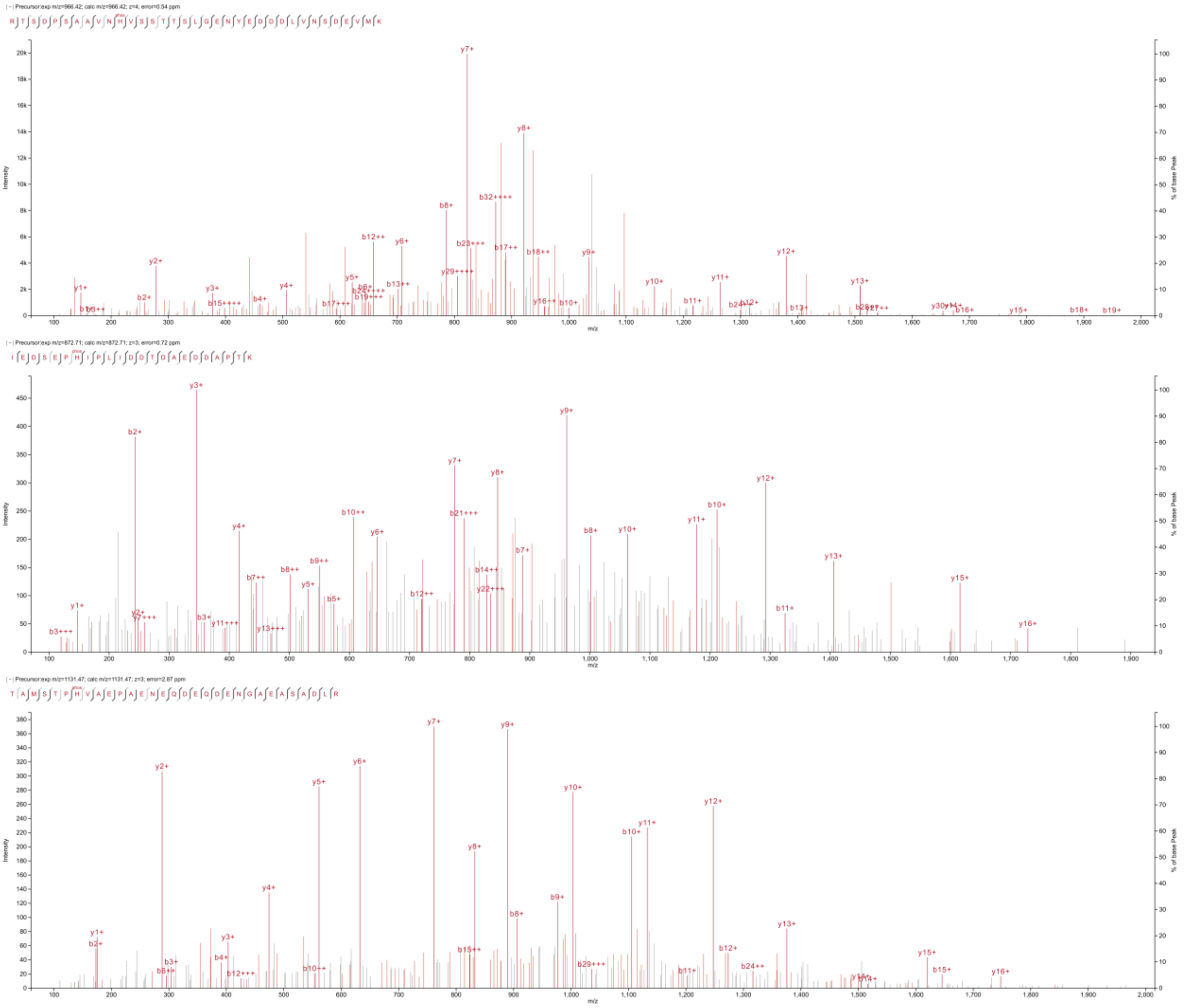

Appendix Figure S6. Example tandem mass spectra for pHis-containing peptides

HCD product ion spectra of (**top**) 4+ ion at *m/z* 966.42: RTSDPSAAVN**pH**VSSTTSLGENYEDDDLVNSDEVMK from Alastin-2 (Q8NHH9); (**middle**) 3+ ion at *m/z* 872.71: IEDSEP**pH**IPLIDDTDAEDDAPTK from Plasma membrane calcium-transporting ATPase 1 (P20020); (**bottom**) 3+ ion at *m/z* 1131.47: TAMSTP**pH**VAEPAENEQDEQDENGAEASADLR from Moesin (P26038).

A **H**NSESESVPSSMFILEDDR

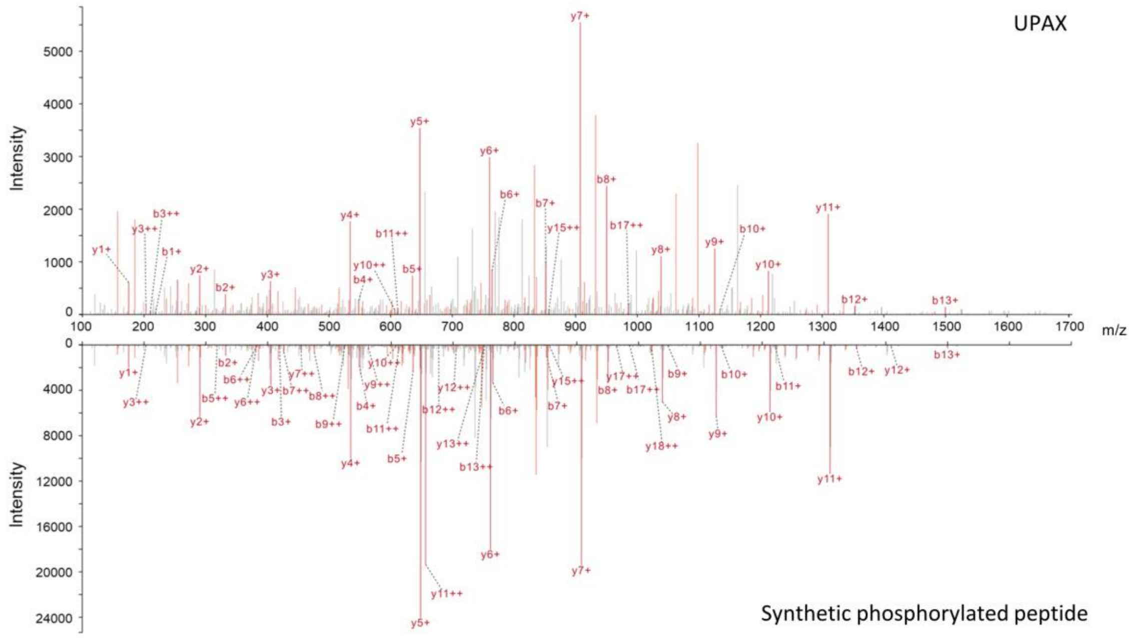

B SESPD**H**MVSQYQQALEEIER

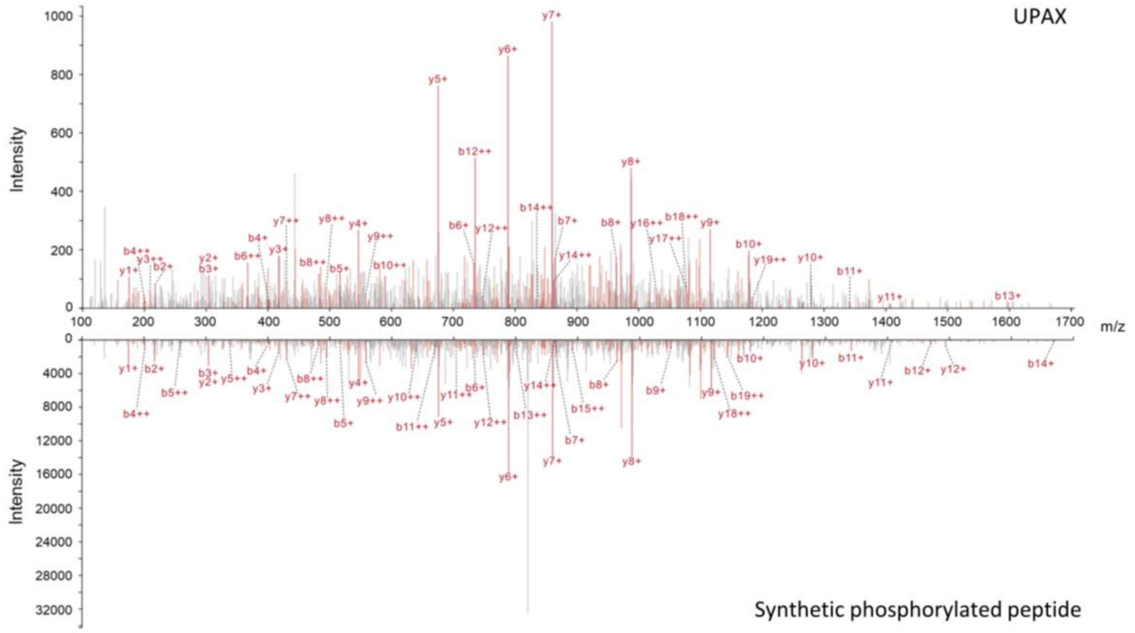

C QA<u>**H**</u>DLSPAAESSSTFSFSGR

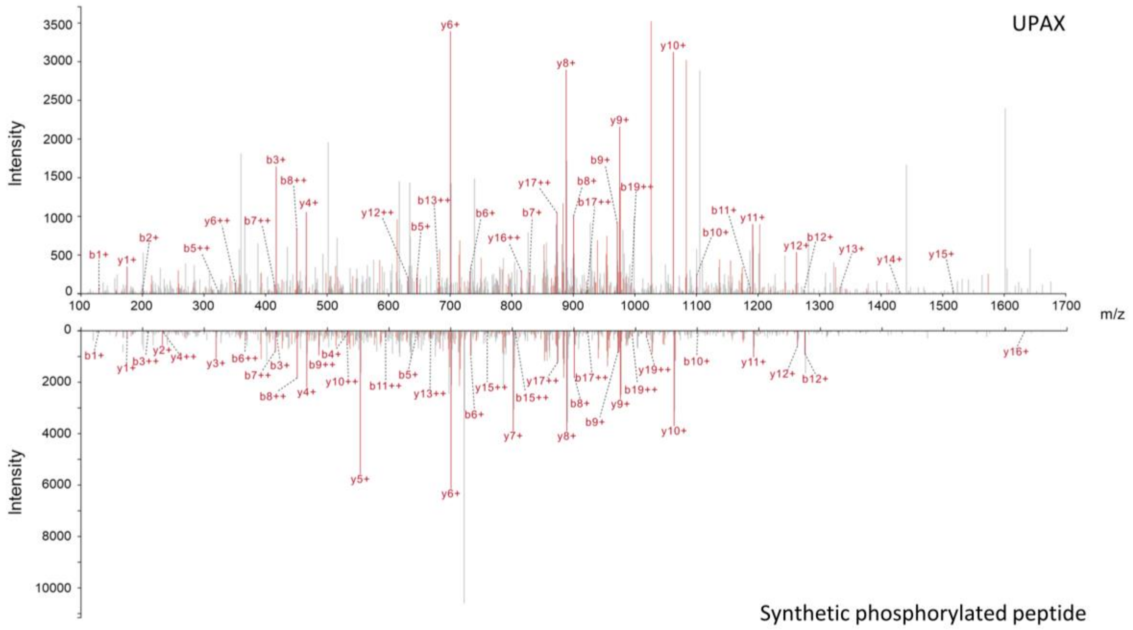

Appendix Figure S7. Spectral comparison of HCD tandem mass spectra of synthetic chemically phosphorylated pHis peptides (bottom) with the analogous pHis-containing spectra from the high-throughput UPAX data (top). (A) doubly charged ion at *m/z* 753.65 pHNSESESVPSSMFILEDDR from RICTOR (Q6R327); **(B)** doubly charged ion at *m/z* 819.36 SESPDpHMVSQYQQALEEIER from MORC3 (Q14149); **(C)** triply charged ion at *m/z* 721.31 from SVIL (O95425). All spectra were manually annotated with the aid of xiSPEC (Kolbowski *et al* 2018). Unlabelled light red peaks are neutral loss-assigned product ions

motif	score	fg.matches	fg.size	bg.matches	bg.size	fold.increase
.......HV......	2.03	5	20	6309	93369	3.70
.......H.....G.	2.31	6	26	6292	99661	3.66
.......HL......	2.76	11	37	12479	112140	2.67

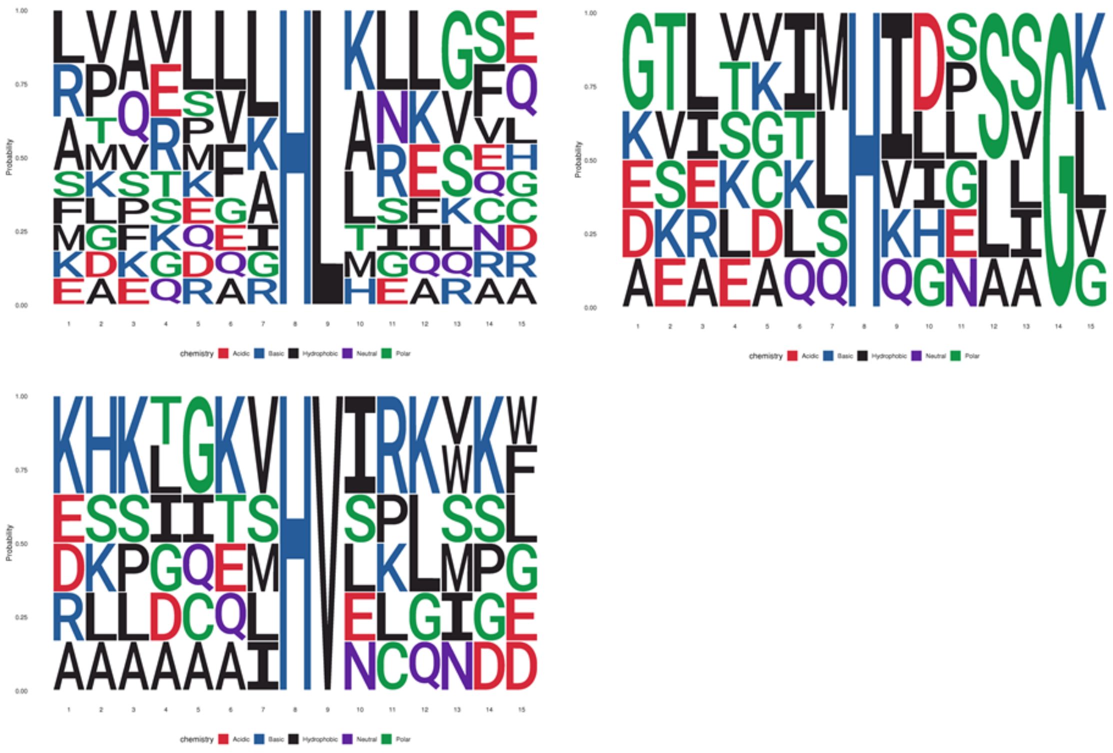

Appendix Figure S8. Motif analysis for pHis-containing peptides. The amino acid sequences surrounding confidently localised sites of pHis (ptmRS ≥ 0.99) were analysed for sequence enrichment using Motif-X (Chou & Schwartz, 2011; Schwartz & Gygi, 2005). Depicted are the sequences of the enriched motifs.

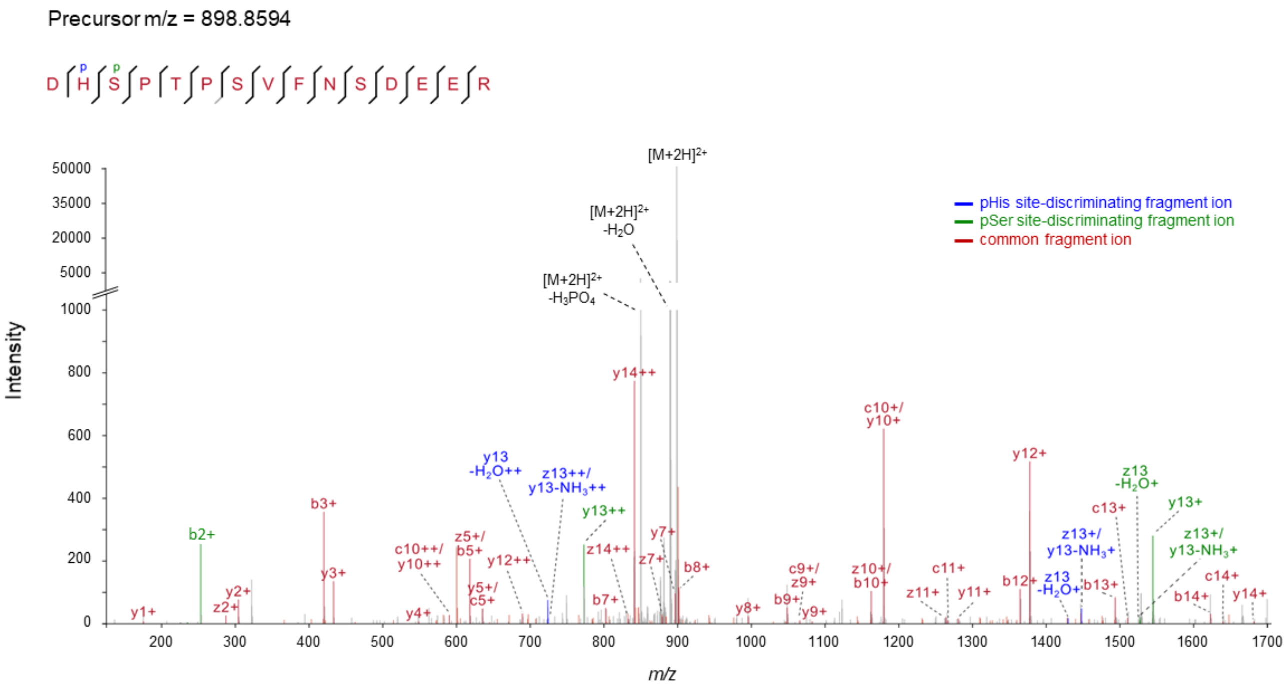

Appendix Figure S9. EThcD product ion mass spectrum of singly phosphorylated DHSPTPSVFNSDEER from FIP1L1. FIP1L1 protein was overexpressed and purified from HEK293T cells, subjected to tryptic digestion and analysed by LC-MS/MS. A precursor ion of m/z 898.8594 corresponding to the doubly charged form of the singly phosphorylated peptide DHSPTPSVFNSDEER was isolated and subjected to EThcD for MS2 analysis in the ion trap. Fragment ions were manually assigned using xiSPEC (Kolbowski *et al* 2018). Site determining ions indicating phosphorylation of either His at position 2 (blue) or Ser at position 3 (green) are annotated, suggesting co-isolation of phosphopeptide isomers and generation of a chimeric spectrum. Common fragment ions are shown in red.

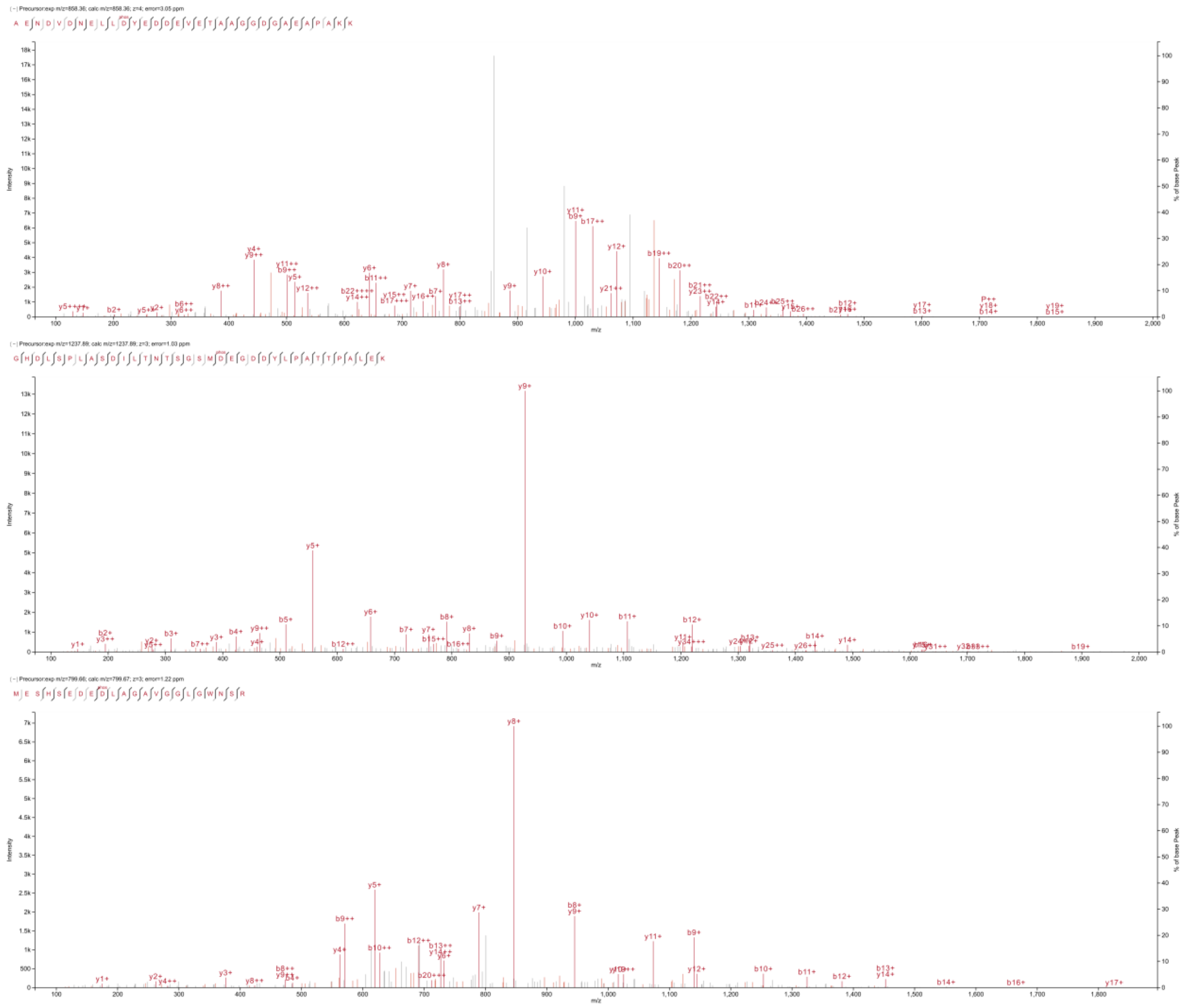

Appendix Figure S10. Example tandem mass spectra for pAsp-containing peptides. HCD product ion spectra of (**top**) 4+ ion at *m/z* 858.36: AENDVDNELL**pD**YEDDEVETAAGGDGAEAPAKK from Spliceosome RNA helicase DDX39B (Q13838); (**middle**) 3+ ion at *m/z* 858.36: GHDLSPLASDILTNTSGSM**pD**EGDDYLPATTPALEK from Microtubule-associated protein 2 (P11137); (**bottom**) 3+ ion at *m/z* 799.66 MESHSEDE**pD**LAGAVGGLGWNSR: from Transcriptional regulator HEXIM2 (Q96MH2).

motif	score	fg.matches	fg.size	bg.matches	bg.size	fold.increase
.......D.....T.	2.12	6	35	8369	165997	3.40
..Q....D.......	2.33	10	85	10535	235806	2.63
.......DI......	1.90	8	55	11379	198999	2.54
.......DP......	2.41	11	75	13071	225271	2.53
.....S.D.......	1.38	6	29	14211	157628	2.29
.....A.D.......	1.76	9	64	13201	212200	2.26
.......DL......	2.23	12	47	21623	187620	2.22
.....L.D.......	1.26	6	23	17597	143417	2.13

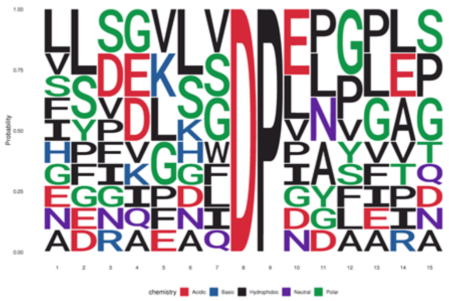

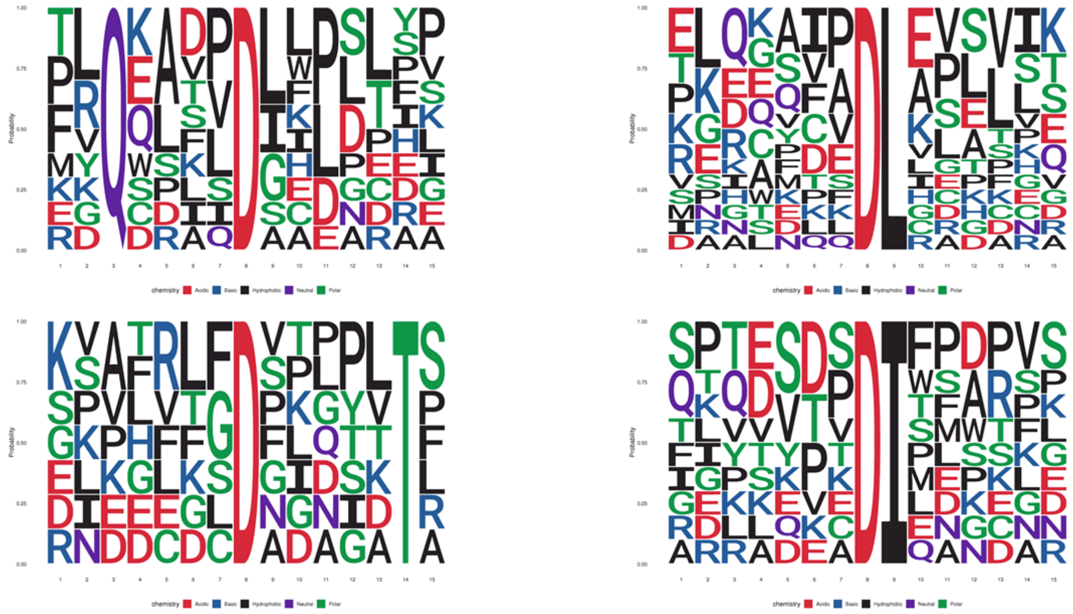

Appendix Figure S11. Motif analysis for pAsp-containing peptides. The amino acid sequences surrounding confidently localised sites of pAsp (ptmRS ≥ 0.99) were analysed for sequence enrichment using Motif-X (Chou & Schwartz, 2011; Schwartz & Gygi, 2005). Depicted are the sequences of the enriched motifs.

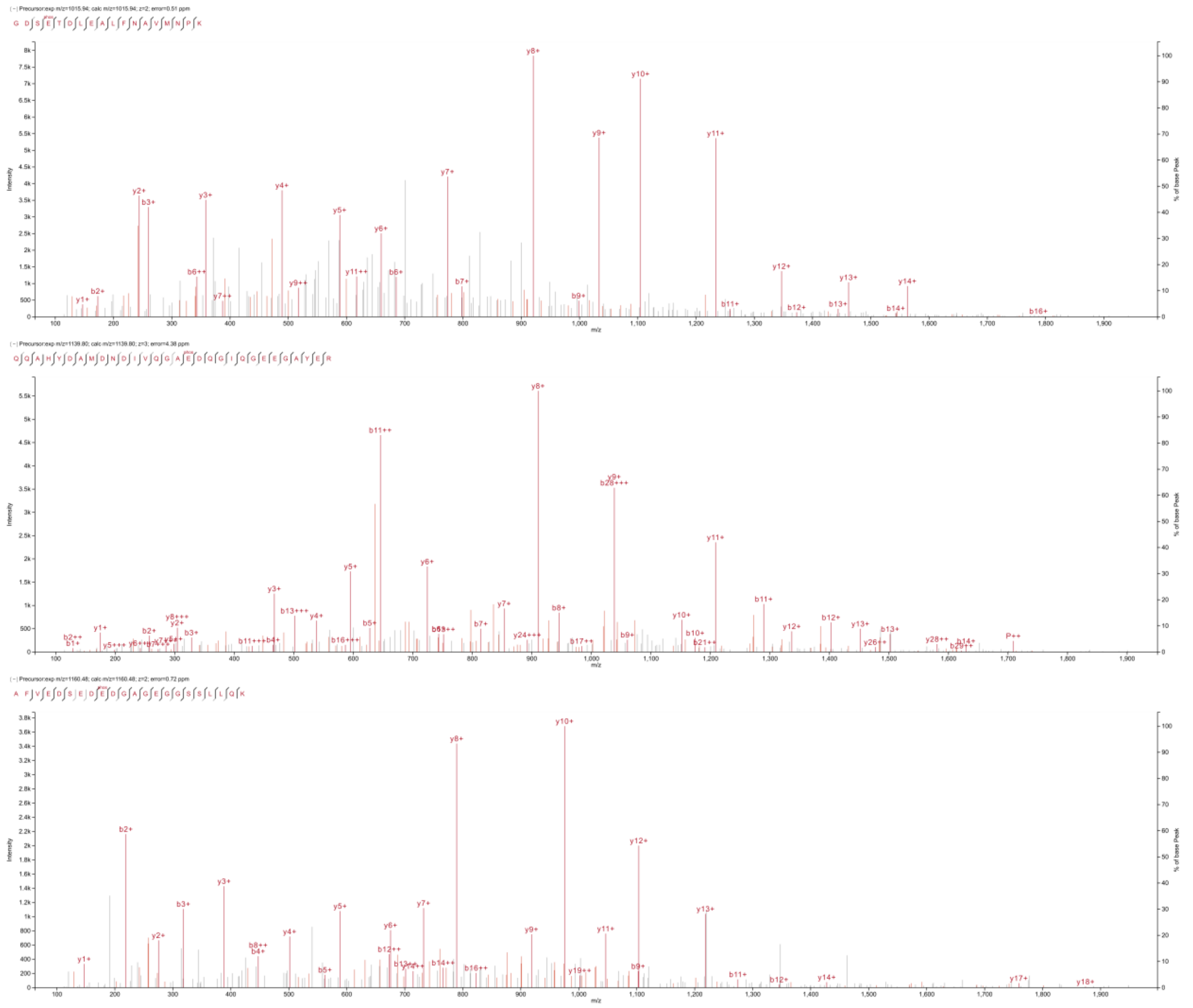

Appendix Figure S12. Example tandem mass spectra for pGlu-containing peptides. HCD product ion spectra of (**top**) 2+ ion at *m/z* 1015.94: GDS**pE**TDLEALFNAVMNPK from Transcriptional coactivator YAP1 (P46937); (**middle**) 3+ ion at *m/z* 1139.80: QQAHYDAMDNDIVQGA**pE**DQGIQGEEGAYER from Golgi integral membrane protein 4 (O00461); (**bottom**) 2+ ion at *m/z* 1160.48: AFVEDSED**pE**DGAGEGGSSLLQK from Protein KRI1 homolog (Q8N9T8).

motif	score	fg.matches	fg.size	bg.matches	bg.size	fold.increase
KQ.....E.......	5.98	5	70	1077	311522	20.66
.......E....T.G	6.27	5	102	938	353160	18.46
...E...EA......	4.29	5	85	2514	331628	7.76
.......E.Q.....	2.14	5	25	11213	224014	4.00
......NE.......	2.08	5	39	8357	257657	3.95
.......E.L.....	2.10	7	20	26357	212801	2.83
...Q...E.......	1.82	7	46	15636	273293	2.66
.K.....E.......	2.37	10	56	19993	293286	2.62
.......E..E....	2.25	9	34	25286	249300	2.61
......QE.......	2.03	9	65	17159	310445	2.51
.......E.D.....	1.99	10	80	17592	329114	2.34
.......EV......	1.96	12	97	20594	352222	2.12

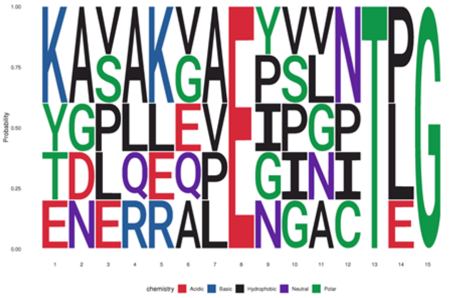

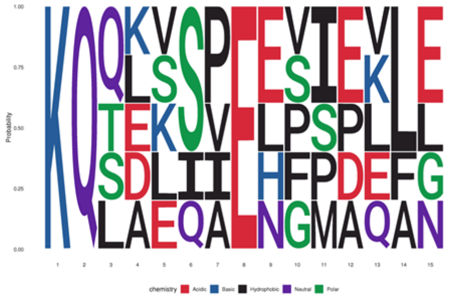

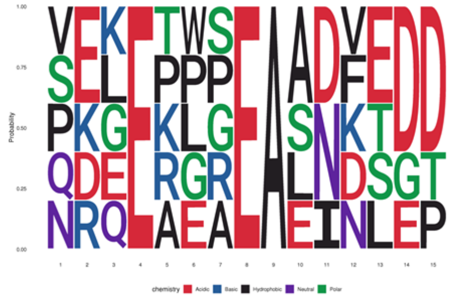

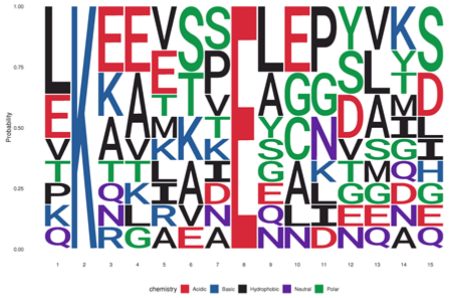

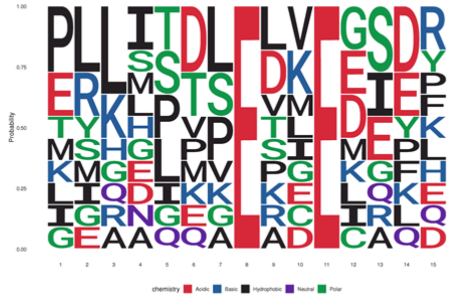

Appendix Figure S13. Motif analysis for pGlu-containing peptides. The amino acid sequences surrounding confidently localised sites of pGlu (ptmRS ≥ 0.99) were analysed for sequence enrichment using Motif-X (Chou & Schwartz, 2011; Schwartz & Gygi, 2005). Depicted are the sequences of the enriched motifs.

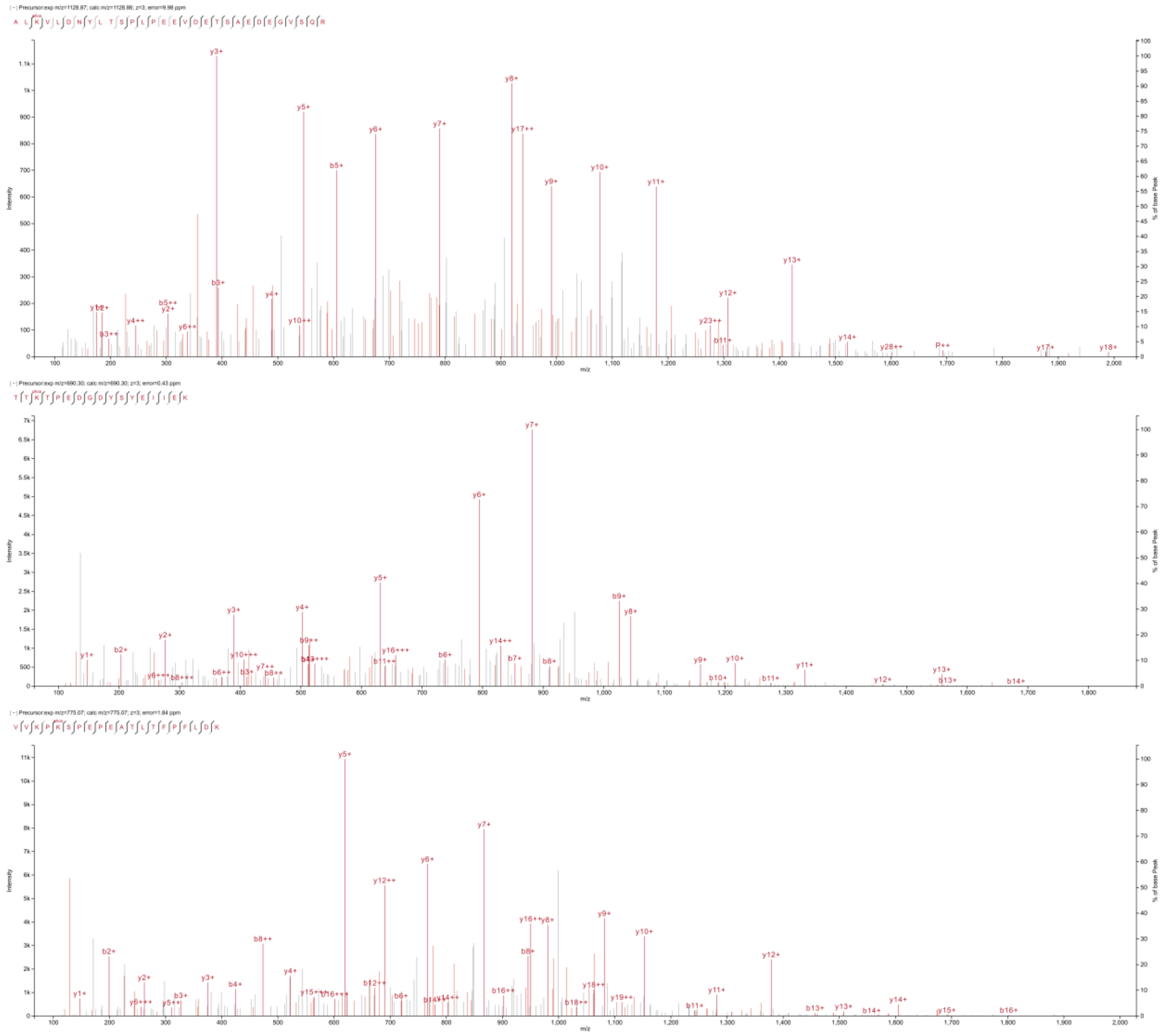

Appendix Figure S14. Example tandem mass spectra for pLys-containing peptides. HCD product ion spectra of (**top**) 3+ ion at *m/z* 1128.87: AL**pK**VLDNYLTSPLPEEVDETSAEDEGVSQR from Chloride intracellular channel protein 1 (O00299); (**middle**) 3+ ion at *m/z* 690.30: TT**pK**TPEDGDYSYEIIEK from Microtubule-associated protein 1B (P46821); (**top**) 3+ ion at *m/z* 775.07: VVKP**pK**SPEPEATLTFPFLDK from LIM and calponin homology domains-containing protein 1 (Q9UPQ0).

motif	score	fg.matches	fg.size	bg.matches	bg.size	fold.increase
.......KP......	2.86	7	34	13118	259115	4.07
.Q.....K.......	2.09	5	27	11717	245997	3.89
..K....K.......	3.04	11	45	23457	282572	2.94
.E.....K.......	1.42	5	22	20416	234280	2.61

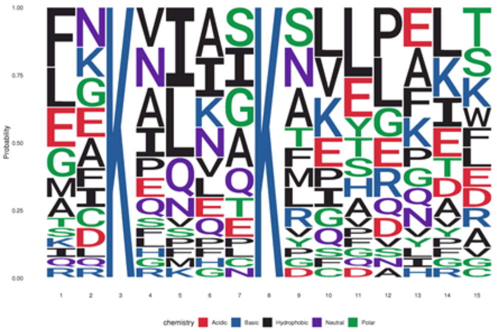

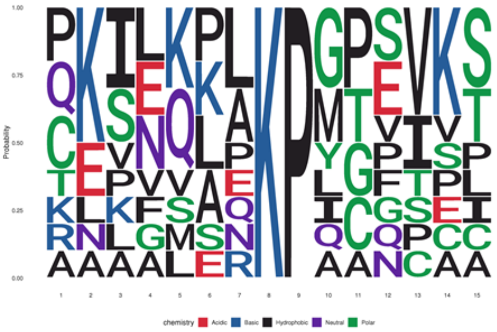

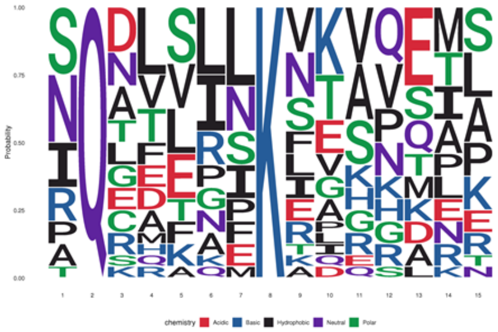

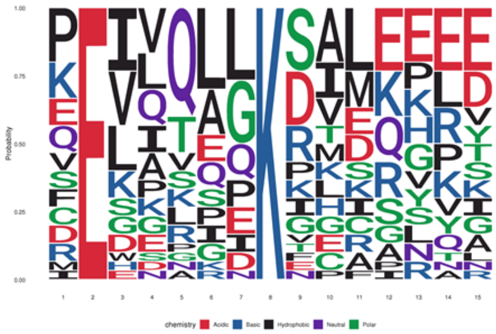

Appendix Figure S15. Motif analysis for pLys-containing peptides. The amino acid sequences surrounding confidently localised sites of pLys (ptmRS ≥ 0.99) not located at the extreme peptide C-terminal residue were analysed for sequence enrichment using Motif-X (Chou & Schwartz, 2011; Schwartz & Gygi, 2005). Depicted are the sequences of the enriched motifs.

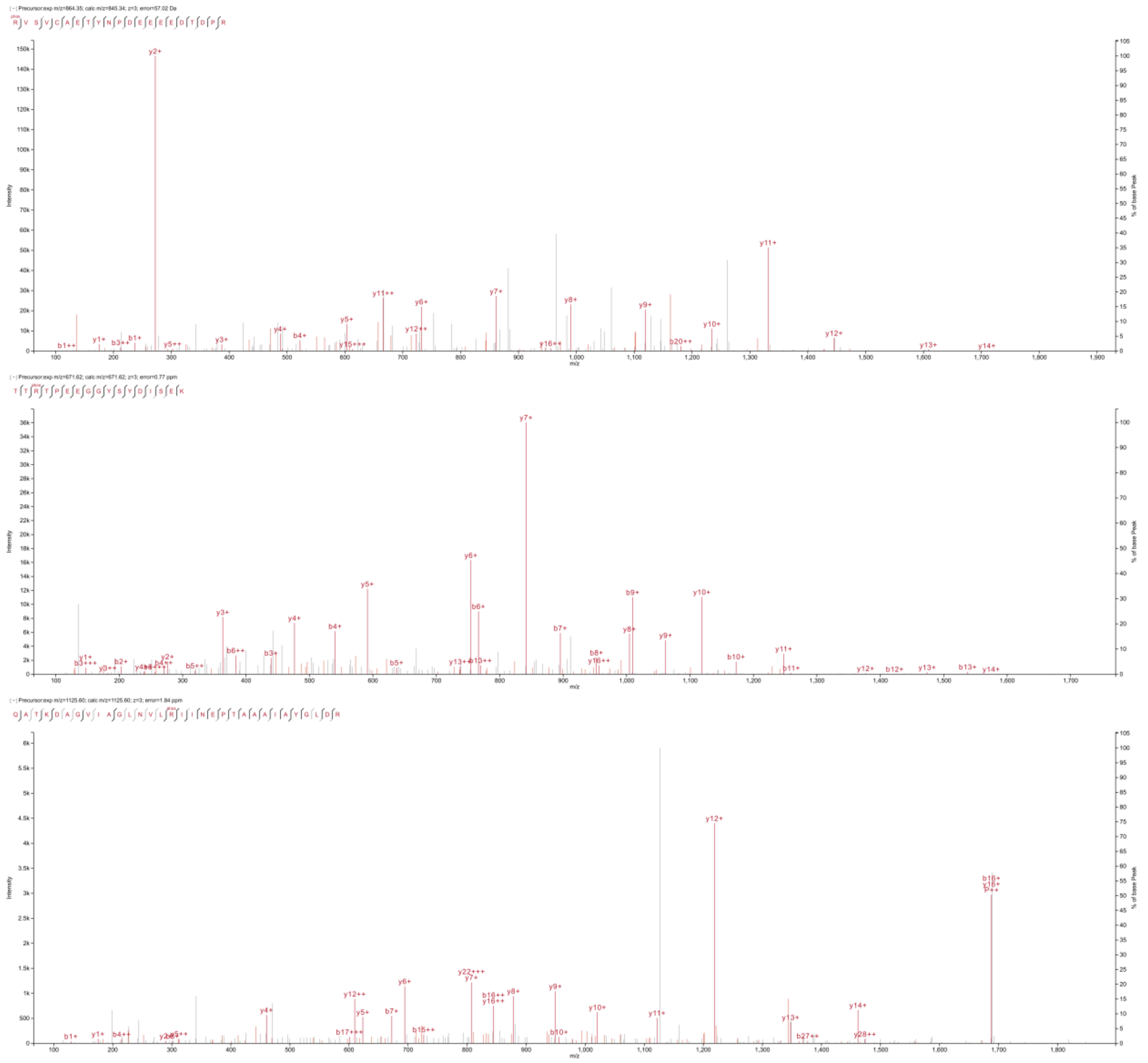

Appendix Figure S16. Example tandem mass spectra for pArg-containing peptides. HCD product ion spectra of (**top**) 3+ ion at *m/z* 864.35: **pR**VSVCAETYNPDEEEEDTDPR from cAMP-dependent protein kinase type II-alpha regulatory subunit (P13861); (**middle**) 3+ ion at *m/z* 671.62: TT**pR**TPEEGGYSYDISEK from Microtubule-associated protein 1B (P46821); (**bottom**) 3+ ion at *m/z* 1125.60: QATKDAGVIAGLNVL**pR**IINEPTAAAIAYGLDR from Heat shock 70 kDa protein 1A (P0DMV8).

motif	score	fg.matches	fg.size	bg.matches	bg.size	fold.increase
.......RP......	3.93	10	45	13926	258155	4.12
......IR.......	2.40	6	35	10769	244229	3.89
.......RG......	2.56	7	29	15891	233460	3.55
..E....R.......	1.53	5	22	17791	217569	2.78

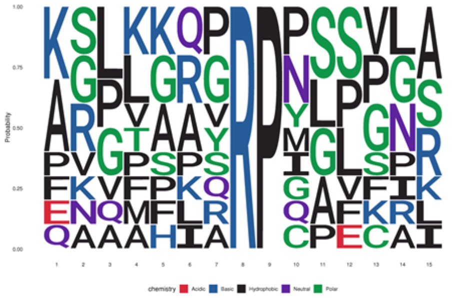

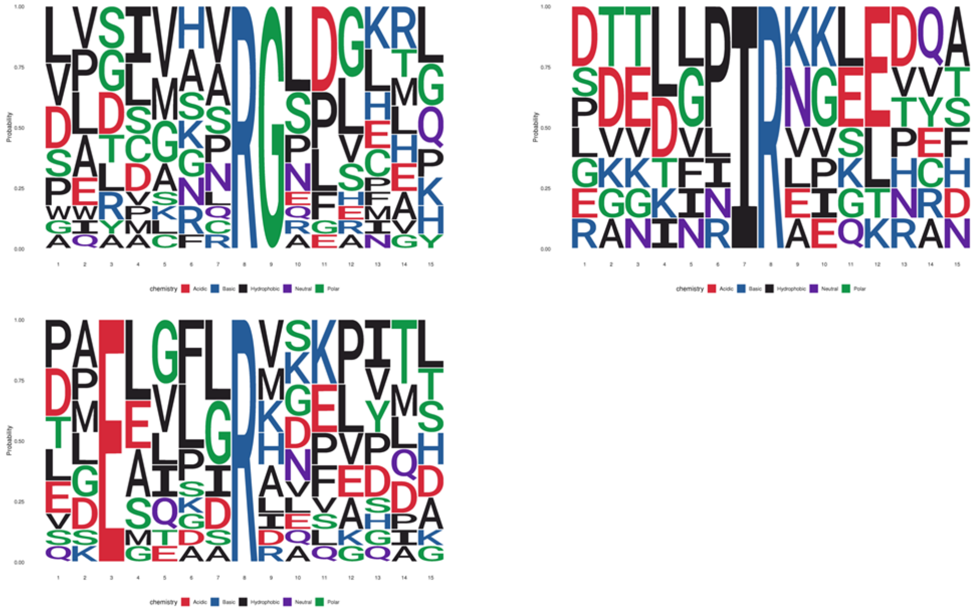

Appendix Figure S17. Motif analysis for pArg-containing peptides. The amino acid sequences surrounding confidently localised sites of pArg (ptmRS ≥ 0.99) not located at the extreme peptide C-terminal residue were analysed for sequence enrichment using Motif-X (Chou & Schwartz, 2011; Schwartz & Gygi, 2005). Depicted are the sequences of the enriched motifs.

motif	score	fg.matches	fg.size	bg.matches	bg.size	fold. increase
E......C.......	2.03	5	20	5771	85212	3.69

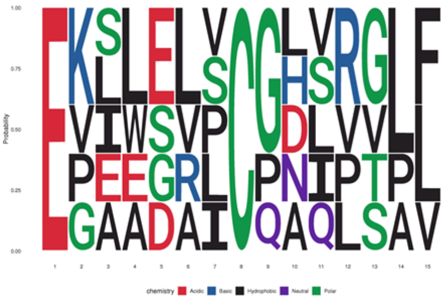

Appendix Figure S18. Motif analysis for pCys-containing peptides. The amino acid sequences surrounding confidently localised sites of pCys (ptmRS ≥ 0.99) were analysed for sequence enrichment using Motif-X (Chou & Schwartz, 2011; Schwartz & Gygi, 2005). Depicted are the sequences of the enriched motifs.

Appendix Tables

Sequence	Site	*m/z*	Charge
**VEADIAG<u>H</u>GQEVLIR**	**His25**	**843.9**	**2^+**
**LFTG<u>H</u>PETLEK**	**His37**	**676.3**	**2^+**
**<u>H</u>GTVVLTALGGILK**	**His65**	**729.9**	**2^+**
**G<u>H</u>HEAELKPLAQSHATK**	**His81**	**645.3**	**3^+**
**GH<u>H</u>EAELKPLAQSHATK**	**His82**	**645.3**	**3^+**
**GHHEAELKPLAQS<u>H</u>ATK**	**His94**	**645.3**	**3^+**
**YLEFISDAII<u>H</u>VLHSK**	**His114**	**655.6**	**3^+**

Appendix Table S1. Phosphohistidine-containing tryptic peptides from myoglobin. Histidine phosphorylated myoglobin was subjected to tryptic digestion and analysed by LC-MS/MS analysis with CID or ETD. Phosphosite localization (shown as underlined residue) was confirmed by manual annotation of spectra.

	Binding	Wash Steps	Elution Steps
**A**	65 % MeCN, 2 % TFA, saturated with glutamic acid, pH 2	*1.* 65 % MeCN, 0.5 % TFA<br>*2.* 65 % MeCN, 0.1 % TFA	*1.* 300 mM NH_3, 50 % MeCN<br>*2.* 500 mM NH_3, 60 % MeCN
**B**	65 mM NH_4OAc, 5 % MeCN, pH 7.5	*1.* 65 % MeCN, 0.5 % TFA<br>*2.* 65 % MeCN, 0.1 % TFA	*1.* 300 mM NH_3, 50 % MeCN<br>*2.* 500 mM NH_3, 60 % MeCN
**C**	3 M lactic acid, 60 % MeCN, 12.5 % AcOH, pH 4	*1.* 2 M lactic acid, 75 % MeCN, 2 % TFA<br>*2.* 2 M lactic acid, 75 % MeCN, 10 % AcOH, pH 4<br>*3.* 80 % MeCN, 10% AcOH	1 % NH_3, 30 mM $(NH_4)_3PO_4$

Appendix Table S2. Conditions evaluated for TiO2 enrichment of pHis (and other) phosphopeptides. Binding, sequential washing and sequential elution buffers for each of the three conditions (A, B and C) used to assess suitability of TiO2 enrichment specifically for pHis-containing peptides are detailed. Different wash and elution solutions were evaluated for each of the three binding conditions. All % are (v/v).

	% Binding pHis peptides	% Elution pHis peptides	% Recovery non-phosphorylated His peptides
A	95 - 100 %	0 %	54 - 133 %
B	98 - 100 %	0 %	7 - 164 %
C	96 - 100 %	0 %	18 - 109 %

Appendix Table S3. Enrichment of pHis peptides by TiO2 fails due to acid-induced hydrolysis of pHis. Amount of non-phosphorylated histidine-containing peptides recovered after TiO_2 enrichment is greater than in the original start material. Range of values for binding and elution/recovery represent analysis from multiple pHis peptides for 2 replicate experiments. Conditions A, B and C for enrichment are detailed in Table S2.

Bound pHis-containing peptides	Elution pH	Eluted pHis-containing peptides	Total recovery of all His-containing peptides
83 - 96 %	pH 7.8	0 %	4 - 18 %
83 - 96 %	pH 7.0	0 %	2 - 37 %
83 - 100 %	pH 6.0	0 %	1 - 35 %

Appendix Table S4. Recovery of pHis peptides following hydroxyapatite chromatography. Recovery of all peptides following hydroxyapatite chromatography was poor, irrespective of elution pH; range of values represents recovery of phosphohistidine-containing peptides across at least 2 replicate experiments.

ptmRS ≥ 0.75	pSer		pThr		pTyr		pHis		pAsp		pGlu		pLys		pArg		pCys		pAla	
pAla FLR	21%		35%		41%		51%		63%		79%		54%		28%		51%		100%	
**siRNA**	**NT**	**PHPT1**	**NT**	**PHPT1**	**NT**	**PHPT1**	**NT**	**PHPT1**	**NT**	**PHPT1**	**NT**	**PHPT1**	**NT**	**PHPT1**	**NT**	**PHPT1**	**NT**	**PHPT1**	**NT**	**PHPT1**
**Total pX peptides**	6678	5865	932	827	222	177	175	154	869	742	977	745	182 (433)	169 (411)	209 (294)	194(273)	74	52	356	283
**Total pX peptides**	**12543**		**1759**		**399**		**329**		**1611**		**1722**		**351 (844)**		**403 (567)**		**126**		**639**	
**Unique pX sites**	2653	2432	567	506	154	116	139	127	618	556	694	559	151 (358)	155 (354)	160 (240)	147 (220)	63	48	251	218
**Unique pX sites**	**3636**		**895**		**236**		**225**		**980**		**1068**		**268 (626)**		**278 (419)**		**103**		**404**	

ptmRS ≥ 0.90	pSer		pThr		pTyr		pHis		pAsp		pGlu		pLys		pArg		pCys		pAla	
pAla FLR	13%		26%		28%		36%		61%		79%		41%		23%		38%		100%	
**siRNA**	**NT**	**PHPT1**	**NT**	**PHPT1**	**NT**	**PHPT1**	**NT**	**PHPT1**	**NT**	**PHPT1**	**NT**	**PHPT1**	**NT**	**PHPT1**	**NT**	**PHPT1**	**NT**	**PHPT1**	**NT**	**PHPT1**
**Total pX peptides**	3961	3448	539	489	119	96	90	79	311	278	343	268	89 (269)	72 (244)	100 (149)	91 (133)	43	27	158	105
**Total pX peptides**	**7409**		**1028**		**215**		**163**		**589**		**611**		**161 (513)**		**191 (282)**		**70**		**263**	
**Unique pX sites**	1672	1559	313	280	85	69	78	67	242	231	268	219	80 (232)	73 (221)	76 (122)	74 (115)	33	26	106	82
**Unique pX sites**	**2317**		**489**		**138**		**129**		**410**		**427**		**140 (406)**		**139 (220)**		**55**		**162**	

ptmRS ≥ 0.99	pSer		pThr		pTyr		pHis		pAsp		pGlu		pLys		pArg		pCys		pAla	
pAla FLR	6%		14%		17%		28%		57%		69%		29%		15%		24%		100%	
**siRNA**	**NT**	**PHPT1**	**NT**	**PHPT1**	**NT**	**PHPT1**	**NT**	**PHPT1**	**NT**	**PHPT1**	**NT**	**PHPT1**	**NT**	**PHPT1**	**NT**	**PHPT1**	**NT**	**PHPT1**	**NT**	**PHPT1**
**Total pX peptides**	1706	1459	233	233	46	37	21	24	84	61	84	69	23 (123)	23 (109)	34 (62)	32 (54)	15	10	46	20
**Total pX peptides**	**3165**		**456**		**83**		**45**		**145**		**153**		**46 (232)**		**66 (116)**		**25**		**66**	
**Unique pX sites**	826	733	130	124	31	26	18	21	64	53	68	55	25 (111)	23 (100)	27 (54)	23 (44)	12	10	26	15
**Unique pX sites**	**1105**		**210**		**51**		**37**		**99**		**112**		**45 (191)**		**47 (91)**		**20**		**37**	

Appendix Table S5. Total number of phosphopeptides and unique sites identified (5% PSM FDR) for each phosphorylated residue and the hypothetical pAla residue, according to site localisation confidence (ptmRS score). Numbers are depicted for all replicate samples treated with either non-targeting (NT) siRNA, PHPT1 siRNA, or the combined data from all 6 samples. For pLys and pArg, the number in parentheses is the total number of identified sites/peptides including those localised to the peptide C-terminus; outside of parentheses are the non-C-terminal mapped pLys or pArg sites. pAla estimated false localisation rate (FLR) for each residue (non-C-terminal Lys/Arg) at each ptmRS value is also presented.

Total % at each *ptm*RS	≥0.99		≥0.90		≥0.75	
	UPAX	TiO_2	UPAX	TiO_2	UPAX	TiO_2
**Ser**	58.28%	91.93%	51.07%	89.91%	44.97%	87.55%
**Thr**	11.08%	5.59%	10.78%	6.05%	11.07%	7.21%
**Tyr**	2.69%	1.24%	3.06%	1.15%	2.92%	0.87%
**His**	1.95%	0.00%	2.84%	0.29%	2.78%	0.22%
**Asp**	5.22%	0.00%	9.04%	0.29%	12.12%	0.87%
**Glu**	5.91%	0.00%	9.41%	1.44%	13.21%	1.97%
**Lys**	10.07%	0.62%	8.95%	0.29%	7.74%	0.87%
**Arg**	4.80%	0.62%	4.85%	0.58%	5.18%	0.44%
**Canonical pX**	**72.0%**	**98.8%**	**64.9%**	**97.1%**	**59.0%**	**95.6%**
**Non-canonical pX**	**28.0%**	**1.2%**	**35.1%**	**2.9%**	**41.0%**	**4.4%**

Appendix Table S6. Proportion of phosphorylated residues identified using either UPAX or with a standard TiO2-based phosphopeptide enrichment workflow. All data were acquired using the same LC-MS/MS acquisition and data interrogation parameters as described in methods.

ptmRS ≥ 0.75	pSer		pThr		pTyr		pHis		pAsp		pGlu		pLys		pArg		pCys		pAla	
pAla FLR	17%		37%		45%		58%		54%		72%		65%		34%		81%		100%	
siRNA	NT	PHPT1	NT	PHPT1	NT	PHPT1	NT	PHPT1	NT	PHPT1	NT	PHPT1	NT	PHPT1	NT	PHPT1	NT	PHPT1	NT	PHPT1
Total pX peptides	6073	5378	725	648	166	129	120	97	713	617	783	598	116 (244)	105 (116)	138 (183)	130 (165)	37	25	272	230
Total pX peptides	**11451**		**1373**		**295**		**217**		**1330**		**1381**		**221 (465)**		**268 (348)**		**62**		**502**	
Unique pX sites	2331	2141	386	351	103	75	90	78	490	453	531	422	93 (193)	90 (177)	98 (140)	85 (117)	30	21	174	168
Unique pX sites	**3099**		**586**		**147**		**134**		**779**		**795**		**150 (310)**		**160 (227)**		**44**		**284**	

ptmRS ≥ 0.90	pSer		pThr		pTyr		pHis		pAsp		pGlu		pLys		pArg		pCys		pAla	
pAla FLR	10%		25%		31%		42%		50%		76%		51%		30%		58%		100%	
siRNA	NT	PHPT1	NT	PHPT1	NT	PHPT1	NT	PHPT1	NT	PHPT1	NT	PHPT1	NT	PHPT1	NT	PHPT1	NT	PHPT1	NT	PHPT1
Total pX peptides	3634	3173	419	385	83	64	56	35	247	217	247	195	52 (133)	39 (107)	59 (85)	51 (66)	21	13	122	86
Total pX peptides	**6807**		**804**		**146**		**91**		**464**		**442**		**91 (240)**		**110 (151)**		**34**		**208**	
Unique pX sites	1489	1393	218	193	50	42	47	33	189	178	179	149	46 (112)	37 (92)	39 (64)	35 (49)	15	11	70	64
Unique pX sites	**2005**		**319**		**79**		**70**		**316**		**282**		**72 (181)**		**67 (103)**		**23**		**111**	

ptmRS ≥ 0.99	pSer		pThr		pTyr		pHis		pAsp		pGlu		pLys		pArg		pCys		pAla	
pAla FLR	4%		12%		18%		40%		50%		76%		41%		27%		45%		100%	
siRNA	NT	PHPT1	NT	PHPT1	NT	PHPT1	NT	PHPT1	NT	PHPT1	NT	PHPT1	NT	PHPT1	NT	PHPT1	NT	PHPT1	NT	PHPT1
Total pX peptides	1564	1359	181	174	32	23	10	7	55	44	47	43	9 (46)	10 (39)	14 (26)	16 (24)	7	4	37	16
Total pX peptides	**2923**		**355**		**55**		**17**		**99**		**90**		**19 (85)**		**30 (50)**		**11**		**53**	
Unique pX sites	733	670	88	80	18	14	9	6	41	36	33	33	9 (41)	10 (36)	9 (21)	7 (14)	5	3	16	11
Unique pX sites	**970**		**132**		**28**		**15**		**64**		**58**		**18 (73)**		**15 (32)**		**6**		**23**	

Appendix Table S7. Total number of phosphopeptides and unique sites identified (1% PSM FDR) for each phosphorylated residue and the hypothetical pAla residue, according to site localisation confidence (ptmRS score). Numbers are depicted for all replicate samples treated with either non-targeting (NT) siRNA, PHPT1 siRNA, or the combined data from all 6 samples. For pLys and pArg, the number in parentheses is the total number of identified sites/peptides including those localised to the peptide C-terminus; outside of parentheses are the non-C-terminal mapped pLys or pArg sites. pAla estimated false localisation rate (FLR) for each residue (non-C-terminal Lys/Arg) at each ptmRS value is also presented.

pAla decoy estimated pX specific FLR: worked example for ptmRS ≥ 0.90

NU_PSMs_{Ala}	**51205**
NU_S_{pAla}	**263**
AA_RF	0.005136

Residue	NU_PSMs_x	NU_FLS_x	U_S_x	***CF***	U_FLS_x	FLR_x	TPS_x
A	**51205**	**263**	162	**0.616**	162	1.00	101
C	6618	34	55		21	0.38	34
D	78691	404	410		249	0.61	161
E	106921	549	427		338	0.79	89
H	14530	75	129		46	0.36	83
K internal	18176	93	*140*		58	0.41	82
R internal	10023	51	*139*		32	0.23	107
S	97141	499	2317		307	0.13	2010
T	40045	206	489		127	0.26	362
Y	12288	63	138		39	0.28	99

Appendix Table S8. Worked example demonstrating how False Localisation Rate (FLR) is estimated at ptmRS ≥ 0.90 based on the number of pAla 'identifications'.

NU_PSMs_x = Counts of each amino acid in the non-unique (redundant) set of PSMs for phosphopeptides; NU_FLS_x = The estimate of non-unique false localisation site count, based on the ratio of 263 pAla sites from 51205 alanine amino acids, and the count of each amino acid in the non-unique phosphopeptide PSMs; U_S_x = The counts of unique sites observed in the data for each amino acid; CF = The collapse factor estimating how the number of false positive sites collapse from the non-unique level to the unique level; U_FLS_x = The estimate of unique false localisation sites per amino acid; FLR_x = estimate of false localisation rate per amino acid; TPS_x = The estimate of the count of true positive sites per amino acid.

Phosphorylated Residue	pX immonium ion *m/z*	MS/MS spectra		No. with a phosphoresidue specific immonium ion		% with a phosphoresidue specific immonium ion	
		ptmRS ≥ 0.99	ptmRS ≥ 0.75	ptmRS ≥ 0.99	ptmRS ≥ 0.75	ptmRS ≥ 0.99	ptmRS ≥ 0.75
**Ser**	140.01	3165	12543	9	47	0.3%	0.4%
**Thr**	154.03	456	1759	1	3	0.2%	0.2%
**Tyr**	216.04	83	399	12	39	14.5%	9.8%
**His**	190.04	45	329	0	0	0.0%	0.0%
**Asp**	168.01	145	1611	0	12	0.0%	0.7%
**Glu**	182.02	153	1722	0	17	0.0%	1.0%
**Lys**	181.07	232	844	4	17	1.7%	2.0%
**Arg**	209.08	116	567	2	21	1.7%	3.7%
**Cys**	155.99	25	126	0	0	0.0%	0.0%

Appendix Table S9. Phosphoimmonium ions are not generally indicative of the phosphorylated residue. HCD tandem mass spectra representing unique singly phosphorylated peptides, where the site of phosphorylation was localised at either ptmRS > 0.99 or 0.75, were interrogated for residue-specific phosphoimmonium ions. Listed are the number and percentage of MS/MS spectra that contain the residue-specific phosphoimmonium ion (>5% relative signal intensity). With the exception of pTyr, where the phosphoimmonium ion at m/z 216.04 was observed in up to 14.5% of spectra, the number of spectra with a residue-specific phosphoimmonium ion was typically <2%.

References

Chou MF, Schwartz D (2011) Biological sequence motif discovery using motif-x. *Curr Protoc Bioinformatics* Chapter 13: Unit 13 15-24

Clubbs Couldron AKM, Byrne DP, Eyers PA (in press*)* Analysis of 1- and 3-phosphohistidine (pHis) protein modification using model enzymes expressed in bacteria. In *Methods in Molecular Biology: Histidine Phosphorylation*, Eyers CE (ed). Springer Nature

Fuhs SR, Meisenhelder J, Aslanian A, Ma L, Zagorska A, Stankova M, Binnie A, Al-Obeidi F, Mauger J, Lemke G, Yates JR, 3rd, Hunter T (2015) Monoclonal 1- and 3-Phosphohistidine Antibodies: New Tools to Study Histidine Phosphorylation. *Cell* 162: 198-210.

Kolbowski L, Combe C, Rappsilber J (2018) xiSPEC: web-based visualization, analysis and sharing of proteomics data. *Nucleic Acids Res* 46: W473-W478

Mamone G, Picariello G, Ferranti P, Addeo F. (2010) Hydroxyapatite affinity chromatography for the highly selective enrichment of mono- and multi-phosphorylated peptides in phosphoproteome analysis. *Proteomics* 10, (3): 380-93.

Schwartz D, Gygi SP (2005) An iterative statistical approach to the identification of protein phosphorylation motifs from large-scale data sets. *Nat Biotechnol* 23: 1391-8

Zhang X, Ye J, Jensen ON, Roepstorff P. (2007) Highly Efficient Phosphopeptide Enrichment by Calcium Phosphate Precipitation Combined with Subsequent IMAC Enrichment. *Mol Cell Proteomics* 6 (11): 2032-42.
