# Supplementary material for: Strong anion exchange‐mediated phosphoproteomics reveals extensive human non‐canonical phosphorylation
Source: EMBO J. 2019 Aug 21;38(21):e100847. doi: 10.15252/embj.2018100847 (PMC6826212; doi:10.15252/embj.2018100847)
Supplement: Supplementary file 2 — Expanded View Figures PDF [file EMBJ-38-e100847-s002.pdf]

Expanded View Figures

Figure EV1. Myoglobin is extensively phosphorylated on His following treatment with potassium phosphoramidate (PPA).

A PPA-treated myoglobin preferentially generates N3-phosphohistidine. Serial dilutions of myoglobin and PPA-treated myoglobin were dotted onto nitrocellulose membrane and incubated with either the N1- or N3-phosphohistidine antibody (or secondary antibody only), as indicated. Phosphorylated PGAM and NME1 proteins were also dotted onto the membrane, along with their unphosphorylated forms, as controls for the N1- and N3-pHis antibodies, respectively. PGAM: phosphoglycerate mutase. NME1: nucleoside diphosphate kinase.

B Zero charge state mass spectrum of intact phosphorylated myoglobin. Phosphorylated myoglobin was analysed by direct infusion via nano-ESI into a Synapt G2-*Si* mass spectrometer. The raw mass spectrum was deconvoluted using MaxEnt1. Up to five phosphate groups are observed per intact myoglobin molecule. Also apparent is the haem-bound form of myoglobin containing up to four phosphate groups.

© 2019 The Authors

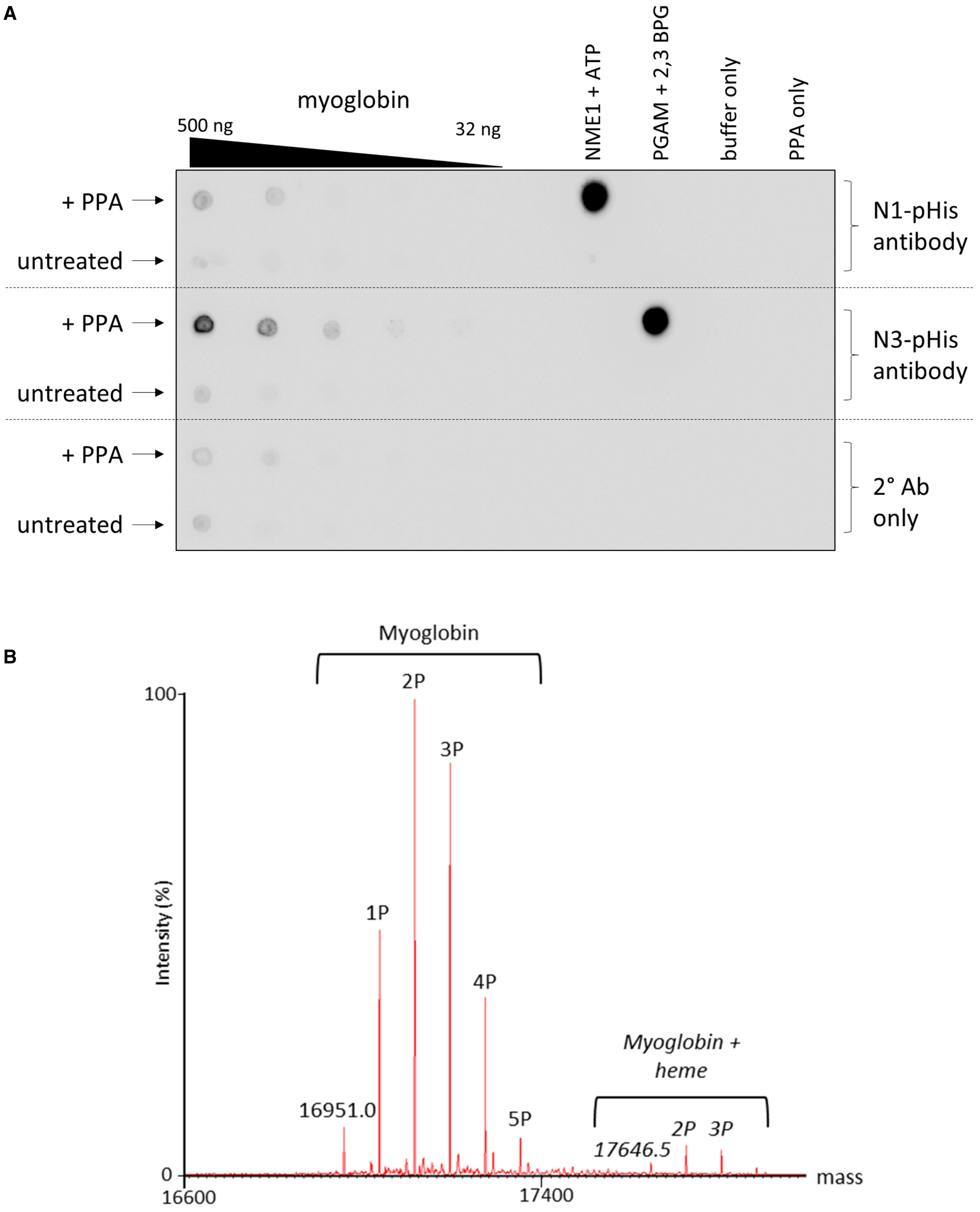

Figure EV1.

 © 2019 The Authors

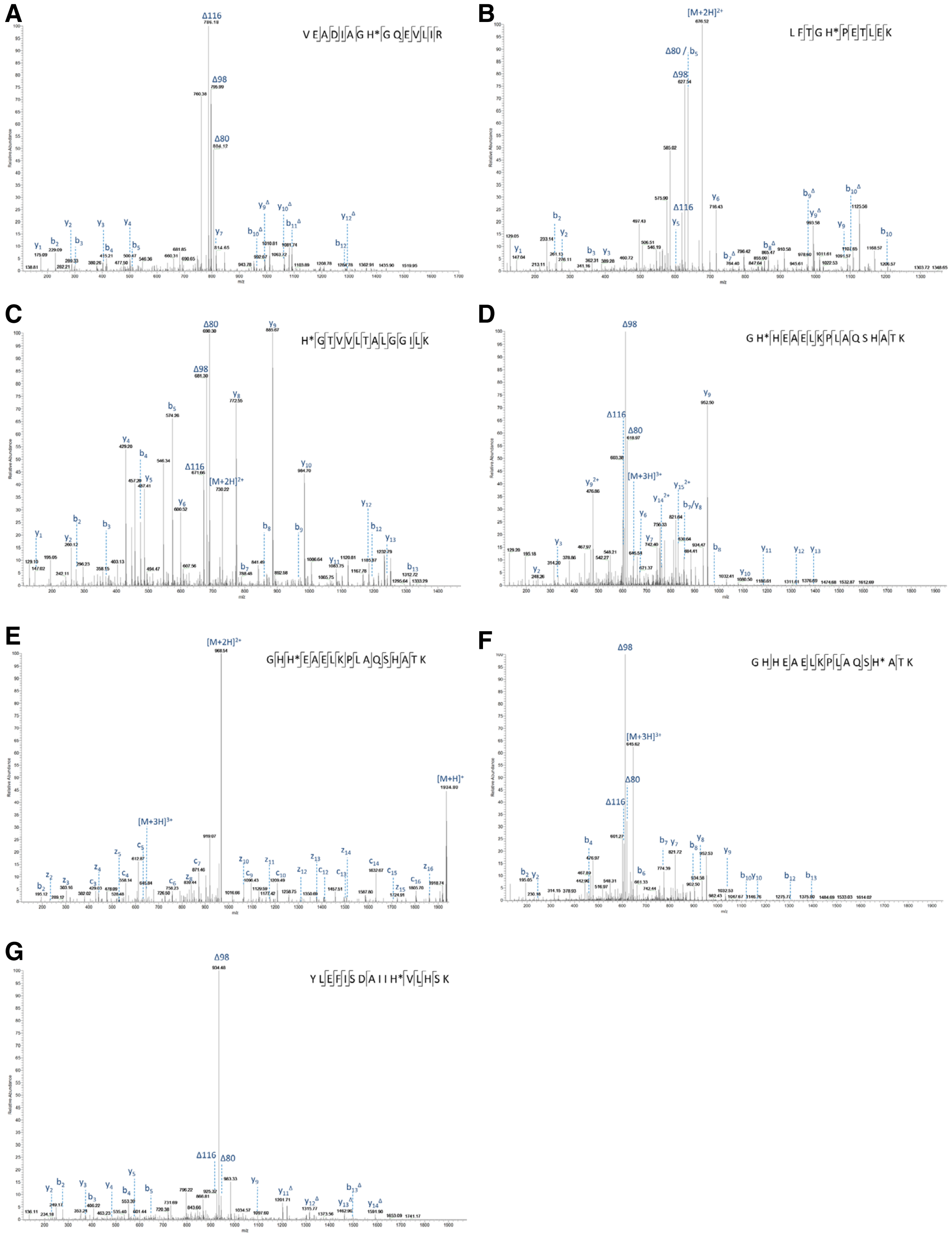

Figure EV2. Product ion spectra generated by HCD (unless stated) of pHis-containing tryptic peptides from equine myoglobin.

A–G The identified phosphorylation site is indicated (*), and the sequence is detailed on the mass spectrum. (A) doubly charged ion at *m/z* 843.9: pHis25; (B) doubly charged ion at *m/z* 676.3: pHis37; (C) doubly charged ion at *m/z* 729.9: pHis65; (D) triply charged ion at *m/z* 645.3: pHis82; (E) ETD spectrum of triply charged ion at *m/z* 645.3: pHis83; (F) triply charged ion at *m/z* 645.3: pHis94; and (G) triply charged ion at *m/z* 655.6: pHis114. The triplet neutral loss ions can be observed in all six HCD spectra (A–D, F–G) for pHis-containing peptides from myoglobin.

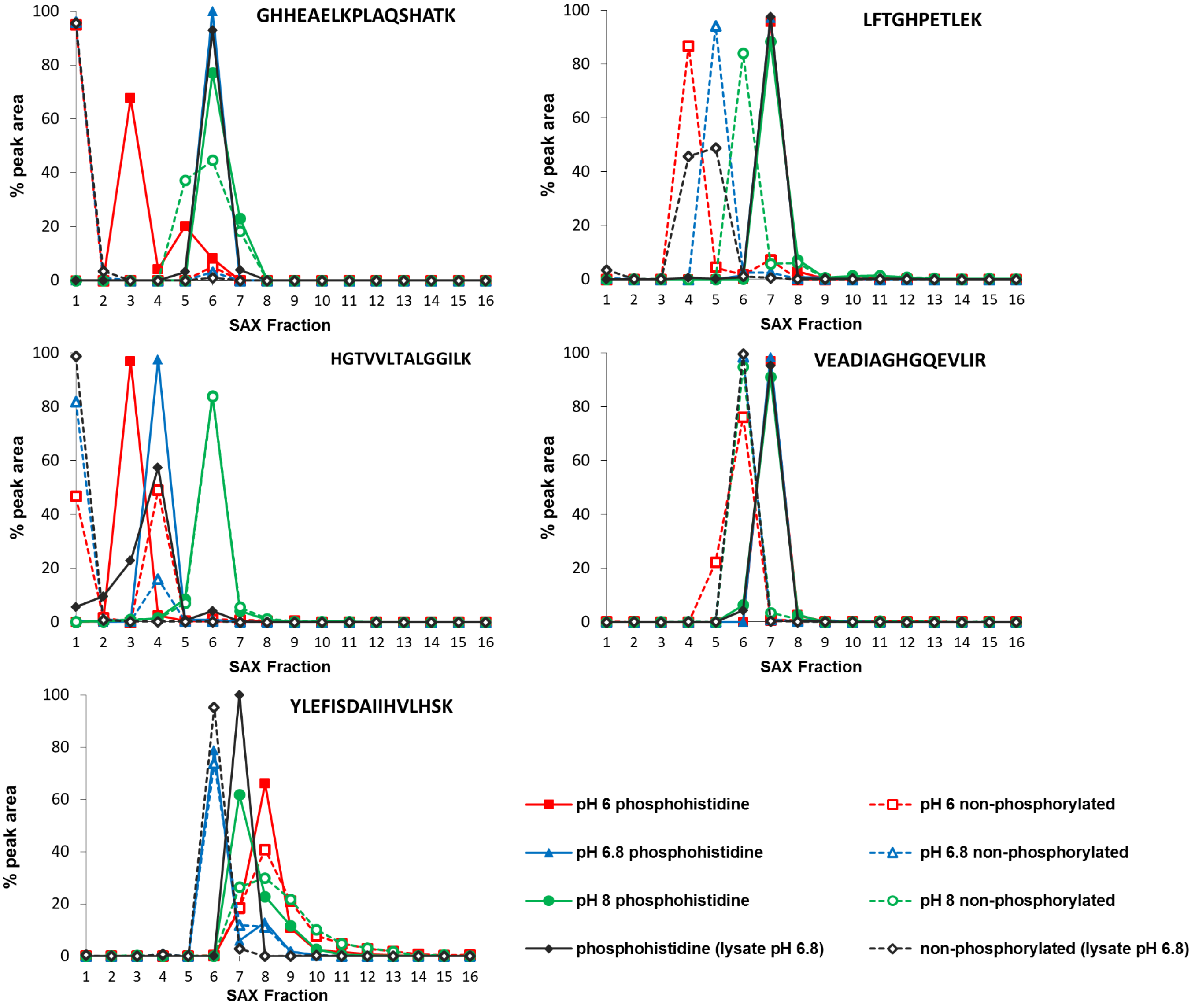

Figure EV3. Optimal separation of pHis myoglobin peptides from their non-phosphorylated counterparts by strong anion exchange (SAX) is achieved at pH 6.8.

Each of the five pHis-containing myoglobin peptides (solid line) and their non-phosphorylated counterparts (dashed line) were quantified across all 16 SAX fractions, where SAX was performed at pH 6 (red), pH 6.8 (blue) or pH 8 (green). Percentage of the total peak area of each individual peptide across the entire SAX separation is plotted for each fraction. SAX at pH 6.8 was also repeated with the phosphorylated myoglobin spiked into a human cell lysate prior to digestion in order to assess pHis stability and effects of SAX separation in a complex protein mixture (black). These data are representative of multiple repeat experiments. As can be seen (middle row, left panel), the non-phosphorylated version of the peptide HGTVVLTALGGILK was quantified at ~50% in both fraction 1 and fraction 4 at pH 6.0, while the phosphorylated peptide is predominantly in fraction 3, confirming pHis hydrolysis during SAX chromatography at this pH. Similar observations were also made for the peptide LFTGHPEYLEK.

 © 2019 The Authors

A

***ptm*RS ≥0.75**	pSer	pThr	pTyr	pHis	pAsp	pGlu	pLys	pArg	pCys
Total pX peptides	11451	1373	295	217	1330	1381	221 (465)	268 (348)	62
Unique pX sites	3099	586	147	134	779	795	150 (310)	160 (227)	44
***ptm*RS ≥0.90**	pSer	pThr	pTyr	pHis	pAsp	pGlu	pLys	pArg	pCys
Total pX peptides	6807	804	146	91	464	442	91 (240)	110 (151)	34
Unique pX sites	2005	319	79	70	316	282	72 (181)	67 (103)	23
***ptm*RS ≥0.99**	pSer	pThr	pTyr	pHis	pAsp	pGlu	pLys	pArg	pCys
Total pX peptides	2923	355	55	17	99	90	19 (85)	30 (50)	11
Unique pX sites	970	132	28	15	64	58	18 (73)	15 (32)	6

B

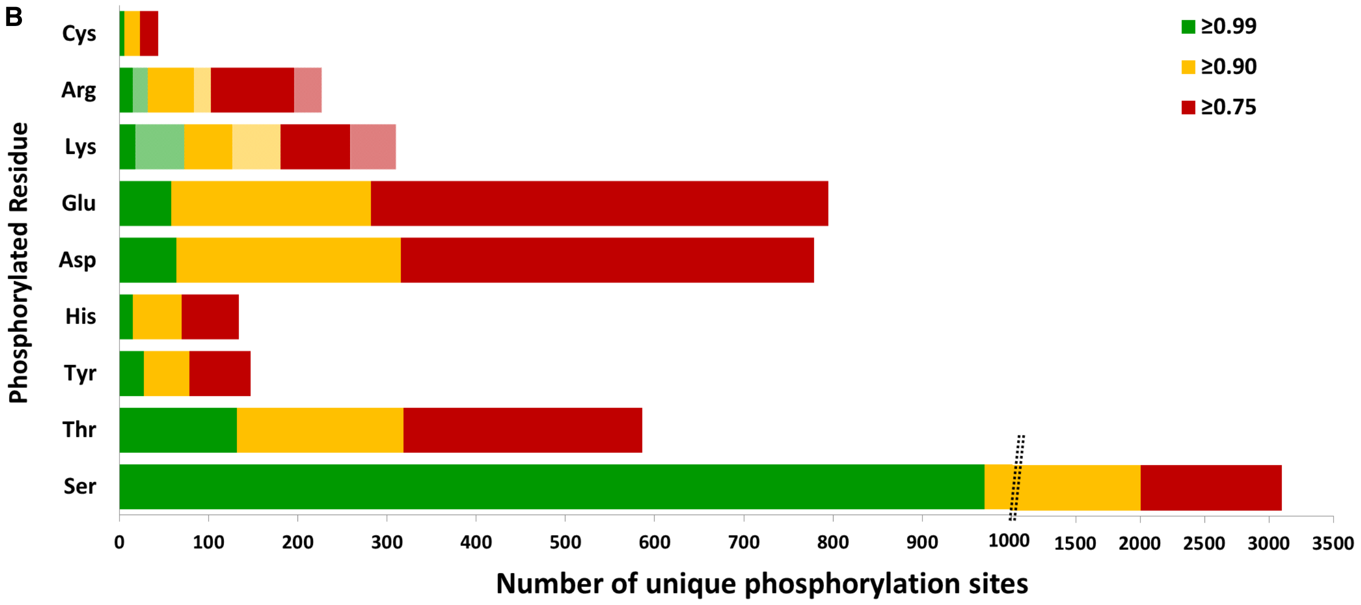

Figure EV4. Total numbers of identified phosphopeptides (1% FDR) and unique phosphosites for each phosphorylated residue according to site localisation confidence (*ptm*RS score).

A For pLys and pArg, the number in parentheses is the total number of identified sites/peptides including those localised to the peptide C-terminus. Non-C-terminally mapped pLys or pArg sites are outside of the parentheses.

B The chart displays the total number of unique phosphorylation sites defined at different site localisation confidence values: *ptm*RS ≥ 0.99 (green); *ptm*RS ≥ 0.90 (yellow); and *ptm*RS ≥ 0.75 (red). Light green, yellow or red indicates those sites of pLys or pArg mapped to the extreme peptide C-terminal residue at each *ptm*RS score cut-off.

© 2019 The Authors
