# Supplementary material for: Strong anion exchange‐mediated phosphoproteomics reveals extensive human non‐canonical phosphorylation
Source: EMBO J. 2019 Aug 21;38(21):e100847. doi: 10.15252/embj.2018100847 (PMC6826212; doi:10.15252/embj.2018100847)
Supplement: Supplementary file 3 — Table EV1 [file EMBJ-38-e100847-s003.docx]

**Expanded View Table**

| **Protein Description** | **Protein** | **Protein Group Accessions** | **Peptide Sequence** | **pHis Site** | **1-pHis/3-pHis** |
| --- | --- | --- | --- | --- | --- |
| **Activity-dependent neuroprotector homeobox protein** | AP1G1 | Q9H2P0 | LMHNASDSEVDQDDVVEWK | H950 | 1-pHis |
| **Centromere protein Q** | CENPQ | Q7L2Z9 | QMHQINSSGVLSLPELSQK | H201 | 3-pHis |
| **Complement component 1 Q subcomponent-binding protein, mitochondrial** | C1QBP | Q07021 | KALVLDCHYPEDEVGQEDEAESDIFSIR | H187 | 1-pHis/3-pHis |
| **DNA replication licensing factor MCM3** | MCM3 | P25205 | DGDSYDPYDFSDTEEEMPQVHTPK | H721 | 3-pHis |
| **Glutamine--tRNA ligase** | QARS | P47897 | GLAYVCHQRGEELK | H359 | 1-pHis |
| **Heat shock protein 105 kDa** | HSPH1 | Q92598 | EEDLEDKNNFGAEPPHQNGECYPNEK | H840 | 1-pHis/3-pHis |
| **Lamin-B1** | LMNB1 | P20700 | TTIPEEEEEEEEAAGVVVEEELFHQQGTPR | H571 | 1-pHis/3-pHis |
| **Nuclear mitotic apparatus protein 1** | NUMA1 | Q14980 | VSLEPHQGPGTPESK | H1995 | 1-pHis |
| **Putative RNA-binding protein 15** | RBM15 | Q96T37 | HCAPSPDRSPELSSSR | H666 | 3-pHis |
| **Pyruvate dehydrogenase E1 component subunit alpha, somatic form, mitochondrial** | PDHA1 | P08559 | YHGHSMSDPGVSYR | H292 | 1-pHis/3-pHis |
| **SAFB-like transcription modulator** | SLTM | Q9NWH9 | ISSKSPGHMVILDQTK | H556 | 1-pHis |
| **Spliceosome RNA helicase DDX39B** | DDX39B | Q13838 | HFILDECDK | H192 | 1-pHis/3-pHis |
| **Structural maintenance of chromosomes protein 4** | SMC4 | Q9NTJ3 | HNTAVSQLTK | H513 | 1-pHis |
| **T-complex protein 1 subunit alpha** | TCP1 | P17987 | DDKHGSYEDAVHSGALND | H542 | 1-pHis/3-pHis |
| **Trifunctional enzyme subunit alpha, mitochondrial** | HADHA | P40939 | HLAILGAGLMGAGIAQVSVDK | H363 | 1-pHis/3-pHis |

**Table EV1. Common phosphohistidine-containing proteins identified both in this UPAX study and by Fuhs et al., (2015).** Listed are the pHis peptides identified from those proteins previously identified to contain either 1-pHis or 3-pHis by virtue of immunoprecipitation (Fuhs et al (2015)), detailing the protein description, short protein name, protein group accession number and residue number in the protein. The exact His residue identified as being phosphorylated is underlined in the peptide sequence.
